# Tuning of ZIF-Derived Carbon with High Activity, Nitrogen Functionality, and Yield – A Case for Superior CO_2_ Capture

**DOI:** 10.1002/cssc.201403402

**Published:** 2015-04-27

**Authors:** Srinivas Gadipelli, Zheng Xiao Guo

**Affiliations:** [a] Department of Chemistry, University College London20 Gordon Street, London, WC1H 0AJ (UK)

**Keywords:** adsorption, carbon, carbon storage, metal–organic frameworks, zinc

## Abstract

A highly effective and facile synthesis route is developed to create and tailor metal-decorated and nitrogen-functionalized active microporous carbon materials from ZIF-8. Clear metal- and pyrrolic-N-induced enhancements of the cyclic CO_2_ uptake capacities and binding energies are achieved, particularly at a much lower carbonization temperature of 700 °C than those often reported (1000 °C). The high-temperature carbonization can enhance the porosity but only at the expense of considerable losses of sample yield and metal and N functional sites. The findings are comparatively discussed with carbons derived from metal–organic frameworks (MOFs) reported previously. Furthermore, the porosity of the MOF-derived carbon is critically dependent on the structure of the precursor MOF and the crystal growth. The current strategy offers a new and effective route for the creation and tuning of highly active and functionalized carbon structures in high yields and with low energy consumption.

## Introduction

A new family of highly porous carbon materials and composites derived from metal–organic frameworks [MOFs; MOF-derived carbons (MDCs)] are attracting considerable interest for clean-energy and environmental applications, such as hydrogen purification and storage, electrodes for Li-ion batteries, supercapacitors, (metal-free) oxygen reduction reactions (ORRs), vapor/gas sensing, carbon capture, and gas separation.[1–5] Given the flexibility in the design of a wide variety of precursor MOF structures with well-defined pores, functional framework ligands, and metal centers, recent efforts have been actively focused on obtaining stable (chemically and thermally) carbon structures with hierarchical porosity and active functional groups, including the conversion of the intrinsic metal centers to highly active catalytic oxides. The advantages of MDCs include controllable pore sizes and specific surface areas (SSAs),[2,5,6] and they have been used to template intrinsic metal oxides[3] and extra functionalities[4,5] such as furfuryl alcohol (FA), carbon nitride, dicyandiamide, glucose, polystyrene and foreign metal oxide precursors. In many cases, the templating/caging, pore, and surface properties are defined directly by the MOF precursor.[2,5,6] The other advantage is the simplicity of the synthesis. MDCs and their composites are obtained by carbonization of the MOF with or without an additional functional medium at a temperature up to 1000 °C for a few hours under a protective dry atmosphere. In spite of the extensive work on the utilization of MDCs in clean-energy and environmental processes, there is still a lack of understanding of the carbonization process and the process-dependent structures and properties of the resultant MDCs.[2a,5j,k] For example, zeolitic-imidazolate frameworks (ZIFs),[5] a subfamily of MOFs, are often studied for MDCs without much control of their structural characteristics. Particularly, the effect of the carbonization and process conditions on the creation of optimum structures with active functional groups and the critical role of the residual metal atoms in the resultant carbon are not considered. The ZIFs are made up of nitrogen-rich ligands and undergo decomposition at temperatures above 600 °C. The carbonization temperature is one of the critical parameters that determines the intricacy of the metal and N sites in the carbon structure.

Herein, ZIF-8 was synthesized and tuned carefully into effective nanoporous carbons [ZIF-derived carbons (ZDCs)] without the addition of a secondary carbon source. A detailed systematic study was conducted to probe the carbonization process. ZIF-8 (unit cell formula: Zn[MIM]_2_, MIM=2-methylimidazolate, C_4_N_2_H_5_) nanocrystals and microcrystals can be synthesized readily in large quantities at room temperature from low-cost precursors with water or methanol as the solvent.[5f,^7^] A solid-state synthesis route was also developed, in which ZnO was mixed with N-containing ligands and heated directly to yield ZIFs with only water as the byproduct.[4a] ZIF-8 is commercially available from Sigma–Aldrich and produced readily by continuous processes.[7e,f] In particular, MOFs and ZIFs with well-defined pore structures and highly accessible pore volumes and surface areas have attracted intense study for gas adsorption and storage.[8,9] However, the readily synthesized ZIF-8 with its nanopore cages (≈1.16 nm) and high specific surface area (≈2000 m^2^ g^−1^) developed through the full coordination of Zn sites with N ligands (2-methylimidazolate) shows a relatively low molecular binding energy and uptake capacity, compared with those of other MOFs with coordinatively unsaturated metal centers.[8] Although some of the MOFs exhibit very good CO_2_ adsorption capacities under practical conditions, the chemical stability of these MOFs, that is, whether these solids maintain their structural integrity and function if subjected to the harsh environment of combustion gases, is under debate.[9] Flue gases containing moisture and acid gases (SO_*x*_ and NO_*x*_) may lead to the degradation of the framework stability or self-interpenetration and, thereby, the loss of accessible surface area (e.g., MOF-5 and MOF-74s are highly air- and moisture-sensitive).[9] Therefore, it is important for the potential adsorbents to be stable in the presence of acid gases and they should have high selectivity, binding, and uptake capacities in a suitable pressure range. In this regard, much work is also devoted to the postsynthetic modification of MOFs, such as metal decoration, ligand functionalization, or surface grafting, and their conversion to active microporous carbons.[9b,^10^] Carbons are well-known adsorbents with rigid pores and high chemical resistance. Furthermore, carbon structures with N functional groups and metal centers are attractive for many applications, such as enhancing electronic conduction, reducing the potential of the ORR, energy storage, and surface molecular binding and selectivity.[4,5] Thus, in principle, the carbonization of ZIF-8 with its oxygen-free, N-containing imidazolate framework ligands and volatile Zn metal centers (melting point 419.5 °C and boiling point 907 °C) should offer microporous carbon with very tunable physicochemical structural properties owing to the variation of the N-doping level and metal decoration.

Here, for the first time, a systematic investigation has been performed by using simultaneous thermogravimetry and mass spectroscopy (TG–MS) to probe the carbonization process of ZIF-8, which involves ligand decomposition and metal evaporation, against carbonization temperature, residence time, and heating rate between 600–1100 °C, 0–24 h, and 2–10 °C min^−1^, respectively. The resultant carbons show marked dependences of the porosity and surface-functional properties on the carbonization conditions. For example, highly N-functionalized and Zn-decorated microporous carbon is achieved in high yield at 600–800 °C. A further increase in temperature leads to an enhanced porosity at the expense of N and Zn content as well as the yield of the carbon. Most importantly, we observed a significant difference between the as-synthesized and acid-treated carbon structures, which show very different porosities and N functionalities. Although the powder XRD (PXRD) results seem to suggest framework decomposition, the as-synthesized carbon samples always show some degree of interaction between the N and Zn atoms. Thus, for the first time, we show that the acid treatment to remove pore-decorated residual Zn atoms can yield highly active carbon structures owing to the pyrrolic N functionalities at a carbonization temperature of approximately 700 °C, which is much lower than the often-reported 1000 °C. Therefore, these structures show interesting CO_2_ uptake characteristics. Importantly, clear enhancements of CO_2_ uptake and CO_2_/N_2_ selectivity through N functionalization and the incorporation of Zn metal sites are observed with a high binding energy, similar to those of amine-functionalized and/or open-metal-structured MOFs. By comparison, we show that these carbons synthesized at 700 °C are arguably the best N-functionalized CO_2_ adsorbents among the MOF- or ZIF-derived carbons reported to date, and they also show CO_2_ uptake and binding superior to those of the precursor ZIF-8. Furthermore, a clear dependence of the porosity on the structure and crystallinity of the precursor is demonstrated for nano- and microsized ZIF-8 crystals. The carbonization behavior of ZIF-8 is compared with that of MOF-5.

## Results and Discussion

**Figure 1:**
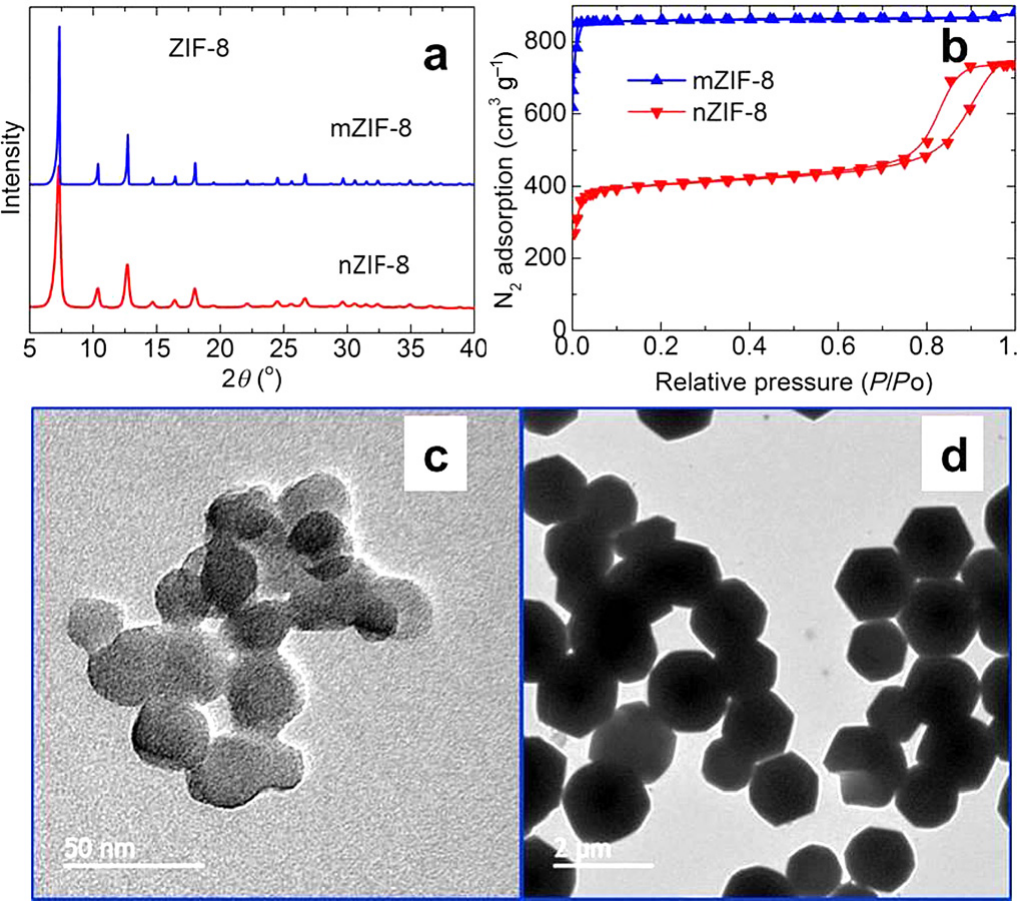
Characterization of nZIF-8 and mZIF-8: a) PXRD patterns, b) N_2_ adsorption–desorption isotherms, and TEM images of c) nZIF-8 and d) mZIF-8. For mZIF-8 in (b), the *y* axis is scaled with 400 cm^3^ g^−1^. The scale bars in c) and d) are 50 nm and 2 μm, and the average particle sizes are 25 nm and 1.5 μm, respectively.

**Figure 2:**
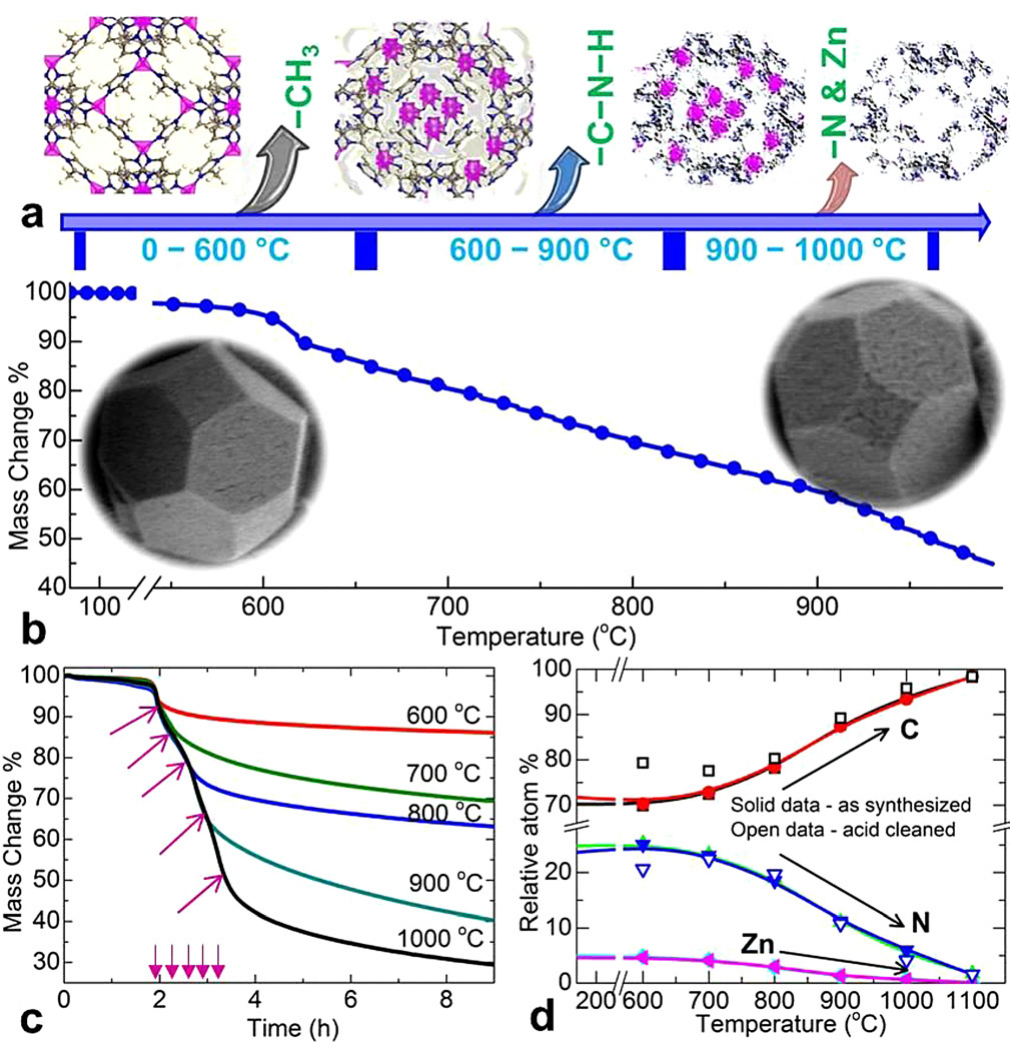
a) Schematic representation of the structural transition of ZIF-8 during controlled carbonization. The atoms in the structure are identified as C grey, H white, N blue, and Zn purple. b) The TGA plot shows the mass loss against heating temperature at 5 °C min^−1^. The insets show SEM images of ZIF-8 and its carbonized product. The first mass loss at approximately 600 °C is attributed primarily to the liberation of =CH_3_ groups, the second gradual mass loss between 600 and 900 °C is due to ligand decomposition and the liberation of =C=N=H mixtures, and the third prominent mass loss is due to the evaporation of Zn. As this carbonization is somewhat slower than those of MOF-5 and MOF-74, no apparent surface microcracks on the crystals are observed. c) TGA isothermal carbonization plots of ZIF-8 at 600–1000 °C with a residence time of up to 7 h; the arrows represent the specific heating time (at 5 °C min^−1^) to reach carbonization temperature. d) XPS relative atomic percentages of C, N, and Zn in the as-synthesized and acid-treated carbons; for better comparison, the adsorbed O is omitted.

As shown in Figures 1 and S2, the PXRD, patterns, 77 K N_2_ isotherms, and TEM images of the ZIF-8 samples reveal highly crystalline and porous nano- (nZIF-8) and microsized (mZIF-8) crystals with BET specific surface areas of 1700 and 1900 m^2^ g^−1^, respectively, in good agreement with the literature values.[5f,^7^] Note that the nano- and microsized crystals show different shapes of N_2_ isotherm, and the tail with further uptake at high relative N_2_ pressure for nZIF-8 is attributed to condensation effects in externally formed pores between the nanoparticles.[7b,d] As shown in Figures 2 and S3, the simultaneous TG–MS analysis shows clearly that the carbonization process of ZIF-8 at ≥600 °C involves the dissociation of the relatively volatile methyl groups from the main structure, followed by the liberation of nitrogen and vaporized zinc metal. Here, it is worth noting that the actual framework decomposition and subsequent carbonization is relatively slow compared with the rapid decomposition of other MOF structures with carboxylate ligands, such as MOF-5 (Figure S4) and MIL-53.[2a] The MS signals reveal that the methyl groups of the imidazolate ligands are the first to dissociate (*m*/*z*=2, 12, 13, 14, and 15) below 500 °C, and the signals related to the ligand nitrogen atoms appear mainly between 600 and 800 °C. Here, it is also worth noting that there is no indication of rapid mass loss above 900 °C, as observed for MOF-5 and MOF-74, corresponding to the simultaneous reduction of ZnO and the vaporization of Zn along with gasification of C (Figure S4).[2a] Thus, the carbonization of ZIF-8 at different temperatures (between 600 and 1100 °C) should yield very different carbon structures.

Therefore, to gain more structural insights into these ZDCs (Figure 2), we performed a prolonged TG–MS carbonizations at five predefined temperatures between 600 and 1000 °C for up to 6 h (Figures 2 c and S5–S9). Clearly, further considerable sample mass loss is observed during the isothermal carbonizations with one-, two-, and three-step decompositions of the ligands at 600–700, 800–900, and 1000 °C, respectively, as is identified through the different rates of thermogravimetric analysis (TGA) sample mass loss as well as one, two, and three peak MS signals for CH_2_ (*m*/*z*=14) and N_2_ (*m*/*z*=28). Increasing N and C decomposition occurs if the sample is carbonized above 800 °C, and the mass loss of the sample nearly doubles. At 600 °C, the majority of the mass loss is attributed to the decomposition of free methyl groups. According to the ZIF-8 formula unit Zn[MeIM]_2_, the release of both methyl groups accounts for a mass loss of 13.2 wt %. Thus, the excess mass loss at ≥700 °C can be attributed directly to the decomposition of the imidazolate ring. On the contrary, the carbonization of MOF-5 (Figure S4) show an abrupt single-step mass loss of over 40 wt % at >500 °C, mainly owing to the decomposition of the framework carboxylate ligands (Figure S10). According to the formula unit, Zn_4_O[BDC]_3_ (BDC=benzene dicarboxylate), the loss of all three equivalents of CO_2_ accounts for a mass loss of 34.3 wt %. The mass loss of over 40 wt % represents a partial evolution or decomposition of the benzene rings (Figure S10). No further mass loss is detected if the sample is left at 600 °C for up to 7 h. At this stage, a clear conversion of the Zn centers to crystalline ZnO occurred.[2a] At a higher carbonization temperature of 1000 °C, a second sharp mass loss is detected above 900 °C owing to the simultaneous reduction of ZnO with C and the release of Zn vapor and ligand C atoms in the form of CO_2_.[2a] This process is analogous to the chemical activation of carbons and, thus, produces highly microporous carbon free from Zn and ZnO.

**Figure 3:**
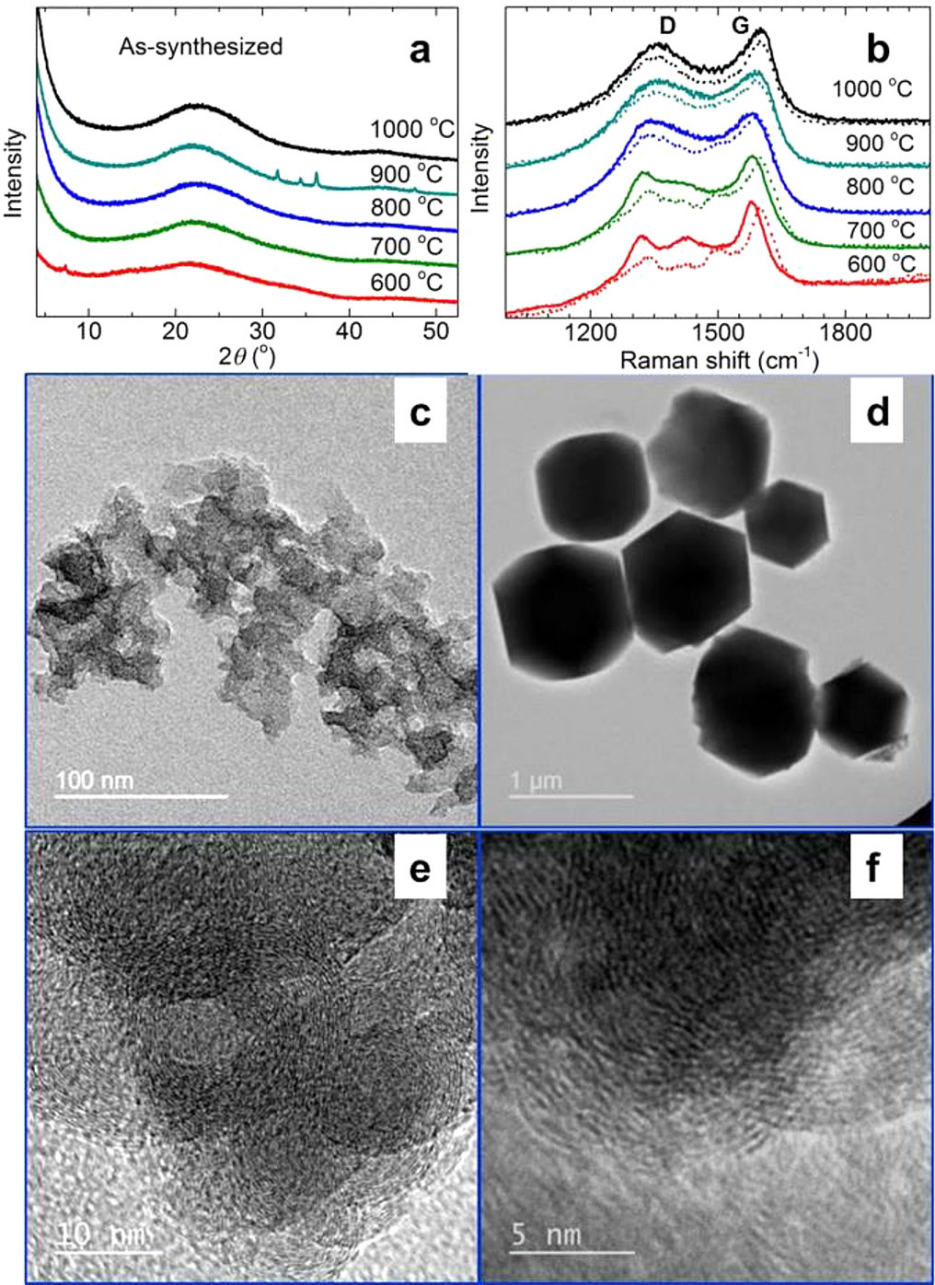
Structural characterization of the as-synthesized ZDCs: a) PXRD patterns and b)  Raman spectra of nZDCs obtained at various carbonization temperatures. TEM images of c, e, f) nZDC1000 and d) mZDC1000; the high-magnification texture of nZDC1000 can be seen in (e) and (f). For easy comparison, the Raman spectra of the acid-treated samples are shown as dotted curves.

Therefore, to clarify the structural features of these ZDCs, we performed the carbonization in a separate tube furnace at six predefined temperatures between 600 and 1100 °C. These as-synthesized carbon samples were divided into two batches, and further structural investigations were performed on the batch of as-synthesized carbons and the batch after acid treatment to remove residual Zn adducts. The variations in the amounts of C, N, and Zn, as detected by X-ray photoelectron spectroscopy (XPS) elemental surveys, relative to the carbonization temperature is shown pictorially in Figure 2 d, which we will discuss in detail below. As shown in Figures 3 a and S11, the PXRD patterns of the nZDCs have weak and broad peaks at 2 *θ* values of 20–27 and approximately 44°, which resemble the diffractions peaks from the (002) and (100) or (101) planes, respectively, of a poorly ordered graphitic structure.[2a,^11^] More or less similar PXRD profiles are seen for all of the nZDCs with variation from a weak graphitic structure at 600 °C to a more graphitic one at 1100 °C. The formation of hexagonal ZnO (e.g., the sharp peaks for the 900 °C sample in Figures 3 a) is seen for the as-synthesized samples if they are left in ambient air for several days. Importantly, no Zn=N complexes are detected in any of the samples.[5i,j] Here, it is worth noting that porous carbon materials with embedded well-crystallized hexagonal ZnO or without any Zn are obtained readily if MOF-5 is carbonized at 600–800 or 1000 °C, respectively (Figure S11 b).

Furthermore, the carbonization is confirmed from the Raman spectra through the clear graphitic D and G bands at $\tilde \nu $
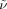
≈1360 and 1600 cm^−1^, respectively (Figures 3 b and S11). Considerably broadened D and G bands and featureless second-order bands (2D and G+D) between $\tilde \nu $
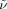
=2700 and 3000 cm^−1^ for all of the samples indicate a disordered carbon network, as evidenced by PXRD.[11,12] The most striking differences are seen between the Raman spectra of the as-synthesized and acid-treated samples and also between those of the samples carbonized below and above 800 °C. All of the acid-treated samples show similar D and G band positions at $\tilde \nu $
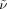
=1340 and approximately 1600 cm^−1^, respectively, akin to those of the disordered graphitic structure. Owing to the N functionalities in the structures, a shift of the G band to higher frequencies by approximately 6 cm^−1^ (blueshift, compared to 1594 cm^−1^ for pure graphitic structure) is observed.[11b,^12^,^13^] The redshifts of the D and G bands of the as-synthesized samples by approximately 20 cm^−1^ to $\tilde \nu $
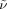
=1324 and 1580 cm^−1^, especially those of the samples carbonized at ≤800 °C, suggest that there is still a definite chemical interaction between the Zn atoms and the N atoms of the imidazolate ligand. Again, no ZnO or Zn–N adducts are detected. In the 600 and 700 °C samples before and after acid treatment, broad bands at $\tilde \nu $
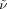
=1425 and 1500 cm^−1^ are assigned to =CH_3_ bending/C=N stretching and ring stretching vibrations, respectively, and suggest that the methyl groups and the ring structure are not lost completely at these temperatures.[13b]

The TEM images shown in Figure 3 support clearly the structural evidence of the carbon samples discussed above. The TEM and SEM images (Figures 3, S2, S12, and S13) also suggest that the carbon samples have similar crystallite shapes and surface structures to those of the precursor ZIFs. As there is no rapid evolution of volatile decomposition products such as CO_2_ as there is for MOF-5 or MOF-74,[2a] the produced carbon samples have mostly crack-free surfaces and do not disintegrate to small particles.

**Figure 4:**
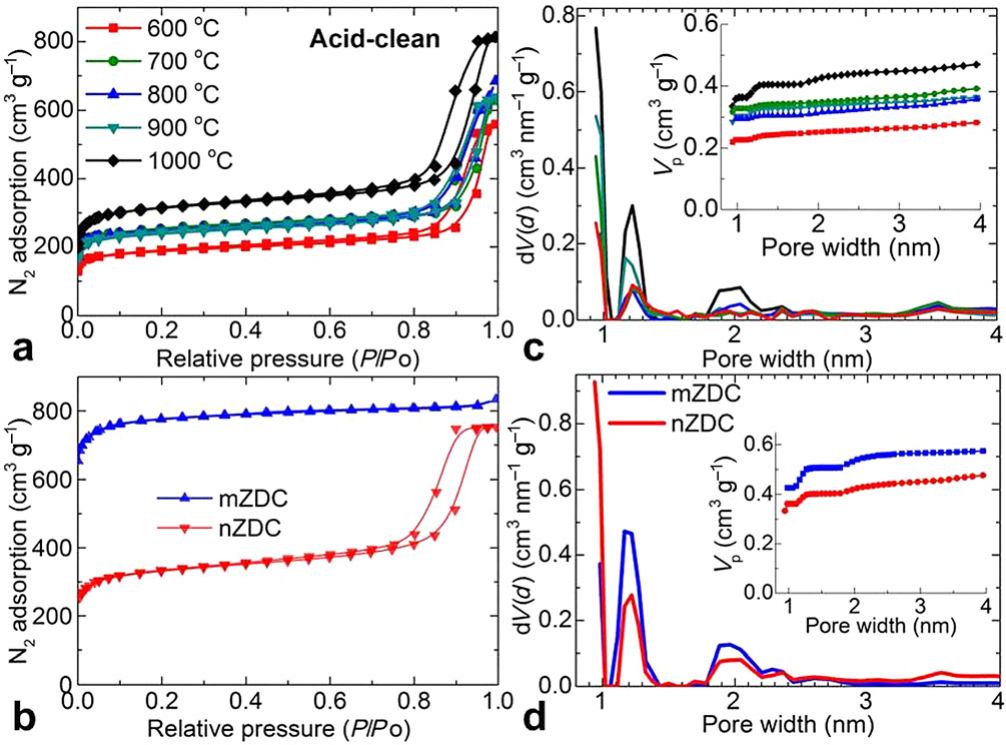
77 K N_2_ adsorption–desorption isotherms of acid-treated a) nZDC*xxx* and b) nZDC1000 and mZDC1000. The corresponding pore size distribution plots are shown in (c) and (d), respectively, and the cumulative pore volume is shown in the inset. The same color codes are applied to identify the carbonization temperatures.

The porosity characteristics of both the as-synthesized and acid-treated carbon samples are shown in Figures 4 and S14. All of the samples show qualitatively similar behavior for their N_2_ isotherms, which are in good agreement with those of the initial ZIF-8. The calculated BET specific surface areas (Table 1), pore size distributions, and pore volume plots, as derived from a nonlocal DFT (NLDFT) model (Figures 4 and S14) show very different pore properties with respect to the carbonization temperature and acid treatment. The transition from the lowest to the highest porosity with increasing carbonization temperature can be attributed directly to the framework-decoupling-assisted migration of the Zn metal centers into the pore cavities and subsequent ligand decomposition, evolution of N, and evaporation of Zn metal (>900 °C; see TG–MS plot in Figures S3 and S5–S9) to create a further microporous carbon network. For example, in the as-synthesized carbon structures, the pore decoration with migrated Zn metal centers can be understood from the smeared-out pore sizes with small pore volume and specific surface area (Figure S14).[5i,j] A sudden pore widening in the samples ≥900 °C suggests the onset of Zn-metal evaporation. Therefore, as shown in Table 1, the nZDC1000 samples before and after acid treatment show similar pore sizes and pore volumes. Furthermore, the reduced porosities (pore volume and specific surface area) of the carbons compared with that of the initial ZIF-8 are attributed to shrinkage of the structure as a result of node Zn migration into pore cavities. This metal center migration and clustering is supported by the formation of crystalline ZnO if the samples are left in air (PXRD pattern in Figure S11).[5j] Importantly, the carbon obtained at 1000 °C shows a comparatively high portion of narrow slit-like pores of less than 1 nm owing to the framework collapse and graphitization; the second major portion of pores have a width of approximately 1.2 nm, which is the same size as the cavities of ZIF-8, and a small fraction of thermally enhanced defect-generated pores have a width of 2 nm, in good agreement with previous reports.[5g,l,n] All of the above porosity, pore size, and pore distribution information suggest that there is a major structural collapse within the regions of the ZIF-8 cavities in the carbons. As shown in Figure 4 d, it is also important to note that the carbonization of microsized crystals of ZIF-8 results in the preservation of a comparatively high portion of ZIF-8 cavities relative to the narrow slit-like pores and, thus, this material shows high porosity. The difference can be attributed primarily to the greater structural order of mZIF-8. A similar dependence of the porosity on the MOF crystal size was also observed for MOF-5; high pore volumes in the carbons derived from millimeter-sized crystals compared with those derived from microsized crystals are attributed to the extended pore paths within the crystals.[2a]

**Table 1:** BET specific surface area (SSA), micropore volume (*V*_m_), room-temperature CO_2_ uptake, and CO_2_/N_2_ selectivity of all the nZDCs before and after acid treatment (represented by a and c) and nZIF-8.

Sample	SSA	*V*_m_	CO_2_ uptake at 25 °C [mmol g^−1^]	*S* 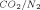		
	[m^2^ g^−1^]	[cm^3^ g^−1^]	0.15 bar	1.0 bar	20.0 bar	
ZIF-8	1700	0.52	0.09	0.70	7.80	11
600^a^	517	0.15	1.08	2.24	4.77	35
700^a^	494	0.17	1.01	2.43	4.40	34
800^a^	616	0.20	1.17	2.78	5.99	30
900^a^	834	0.29	1.24	3.35	8.16	24
1000^a^	1292	0.44	1.04	3.31	10.06	18
600^c^	716	0.25	1.09	2.66	6.48	62
700^c^	950	0.35	1.40	3.51	7.82	79
800^c^	922	0.31	1.22	3.27	7.70	29
900^c^	925	0.34	1.10	3.21	7.80	25
1000^c^	1222	0.43	0.99	3.22	10.21	22

**Figure 5:**
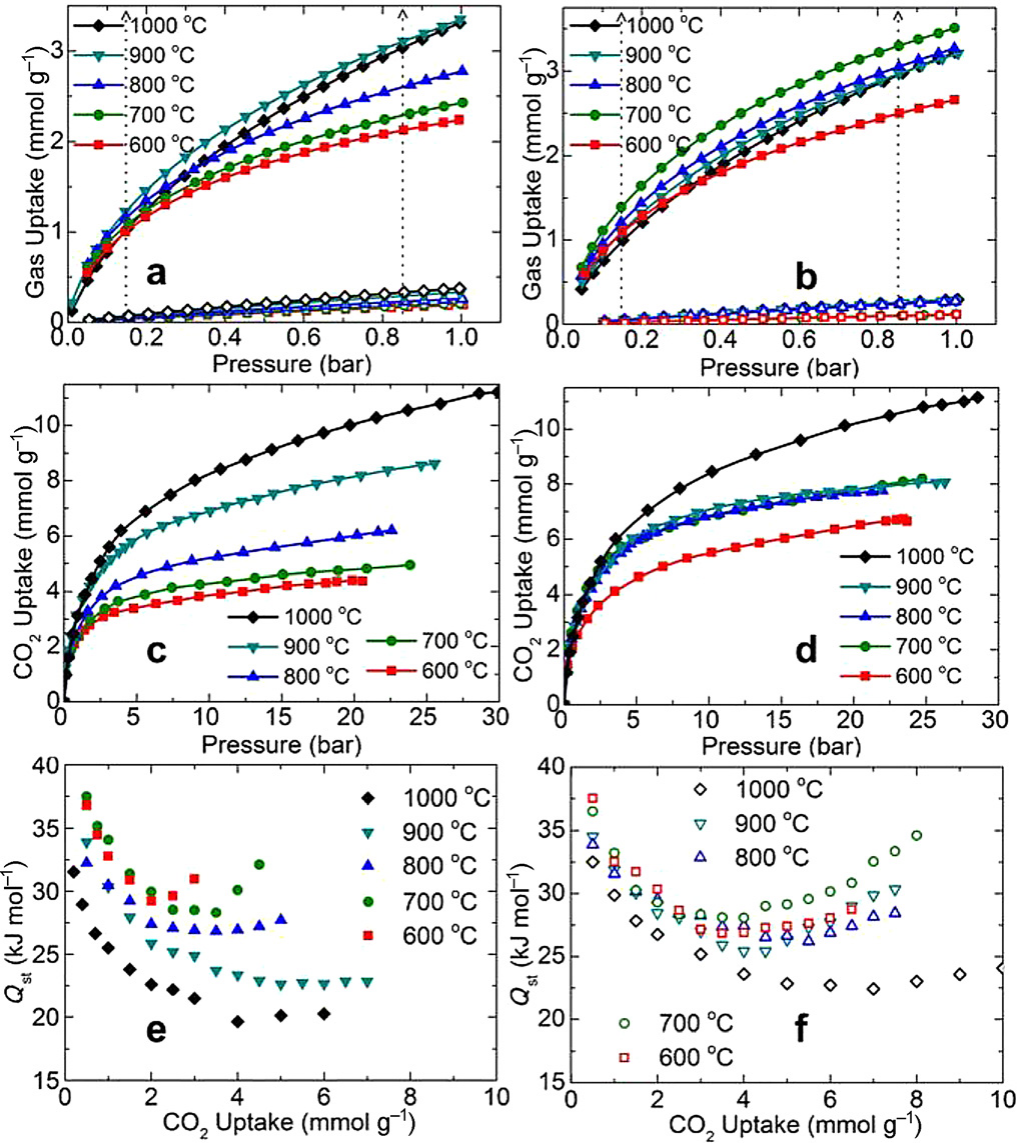
Room-temperature (25 °C) uptake isotherm and isosteric heat of adsorption plots of nZDCs: a, b) low-pressure CO_2_ (solid data) and N_2_ (open data) uptake up to 1 bar, c, d) high-pressure uptake data up to 30 bar, and e, f) isosteric heat of adsorption. All panels on the left and right represent the as-synthesized and acid-treated carbon samples, respectively.

The CO_2_ uptake isotherms of the as-synthesized and acid-treated carbon samples are shown in Figures 5 and S15–S20. Clearly, the high-pressure (up to 30 bar) uptake isotherms of the samples are in good agreement with their porosity characteristics: the CO_2_ uptake shows a more or less linear dependence with the porosity for both the as-synthesized samples and the acid-treated samples (Figure S18). However, as shown in Figure 5 a and b and Figure S18, a different behavior exists for the low-pressure CO_2_ uptakes. For example, the uptake value at pressures of 0.15 bar, equivalent to the partial pressure in the flue gas, and close to 1 bar do not fit the trend of the high-pressure CO_2_ uptakes and porosity variation. For better understanding, the porosity (BET specific surface area and micropore volume) and low- and high-pressure CO_2_ uptake values at 25 °C for all of the carbon samples and the original ZIF-8 are summarized Table 1. Another interesting difference is that if the CO_2_ uptakes are normalized to BET specific surface area and/or pore volume, the as-synthesized samples carbonized up to 900 °C always show higher CO_2_ uptakes than those of the acid-treated samples (Table S1). As Zn metal starts to vaporize above 900 °C, there is not much difference between the as-synthesized sample obtained at 1000 °C and that after acid treatment. Thus, it is conceivable that the residual Zn metal in the carbon pores enhances the CO_2_ uptake.

Similarly, the Zn-free acid-treated samples that possess similar porosities show reduced CO_2_ uptake at the low-pressure region (see Figure 5 b and Table 1) with increased carbonization temperature (700–900 °C). This can be attributed directly to the loss of N content in the structures, as evidenced by the TG–MS results (Figures S3–S9). The overall effects of the narrow pore size and residual Zn metal in the as-synthesized samples as well as the N functionality in the acid-treated carbon samples are also seen in the plots of their isosteric heats of adsorption (*Q*_st_) against CO_2_ uptake (Figure 5 e and f).[5a,^8^,^14^] The *Q*_st_ values of 30–40 kJ mol^−1^ for near-zero coverage are comparable to those of amine-functionalized porous solids or twice those of the many known highly porous pure-phase carbons.[2a,^14^] Similar to the open-metal-enhanced molecular binding in MOFs, Zn-metal-enhanced high *Q*_st_ values are seen for the as-synthesized carbons carbonized below 900 °C.[8,14c,d] The enhanced binding can be attributed to the strong electrostatic interactions between the metal atoms and the CO_2_ quadrupole moment. Owing to the enhanced metal–CO_2_ interaction, one may expect the CO_2_ molecule to be polarized slightly.[8c] A comparison of the two samples with and without Zn (before and after acid treatment, obtained at 600–700 °C) suggests clearly that the Zn-metal-decorated carbons yield higher *Q*_st_ values and normalized CO_2_ uptakes than those of the N-functionalized carbons, as noted in Table S1, with respect to the BET SSAs and pore volumes. The parabolic shape of the *Q*_st_ curves is attributed to the saturation of active sites and the subsequent enhancement of the intramolecular sorbate–sorbate and sorbent–sorbate interactions with increasing CO_2_ pressure.[8,14c] In Table 1, we also show the CO_2_/N_2_ selectivity of all of the samples calculated from the ideal adsorbed solution theory (IAST) model (see Supporting Information) for pure-component CO_2_ and N_2_ isotherms measured at 25 °C (Figure 5 a and b and Figures S19 and S20). For the as-synthesized samples, the Zn-metal-enhanced CO_2_/N_2_ selectivities can be understood from the strengths of the electrostatic interactions and induced polarizabilities. CO_2_ exhibits a higher quadrupole moment and polarizability than N_2_ (−13.71×10^40^ vs. −4.91×10^40^ cm^2^ and 29.1×10^−25^ vs. 17.4×10^−25^ cm^3^, respectively).[8d,e] N_2_ is chemically much more inert and exhibits less polarizability than CO_2_; thus, the N_2_ binding enhancement on the metal is weak. As shown in Table 1, the acid-treated samples carbonized at 600 and 700 °C show the highest CO_2_/N_2_ selectivities, which are twice those of their as-synthesized counterparts and notably three to four times higher than that of the sample carbonized at 1000 °C. All of the above experimental findings suggest that there is still considerable Zn=N coordination in the carbon samples obtained at ≤700 °C; therefore, the acid treatment to remove Zn metal leaves highly active N atoms in the carbon network to enhance the CO_2_ uptake and selectivity over N_2_.[15] Similarly, as listed in Table S2, the carbons with highly active N atoms show relatively high capacities for vacuum swing adsorption (VSA), whereas the high-temperature-carbonized samples with high surface areas show good performance for pressure swing adsorption (PSA; Figure S18 c).[2a,8a,14c]

**Figure 6:**
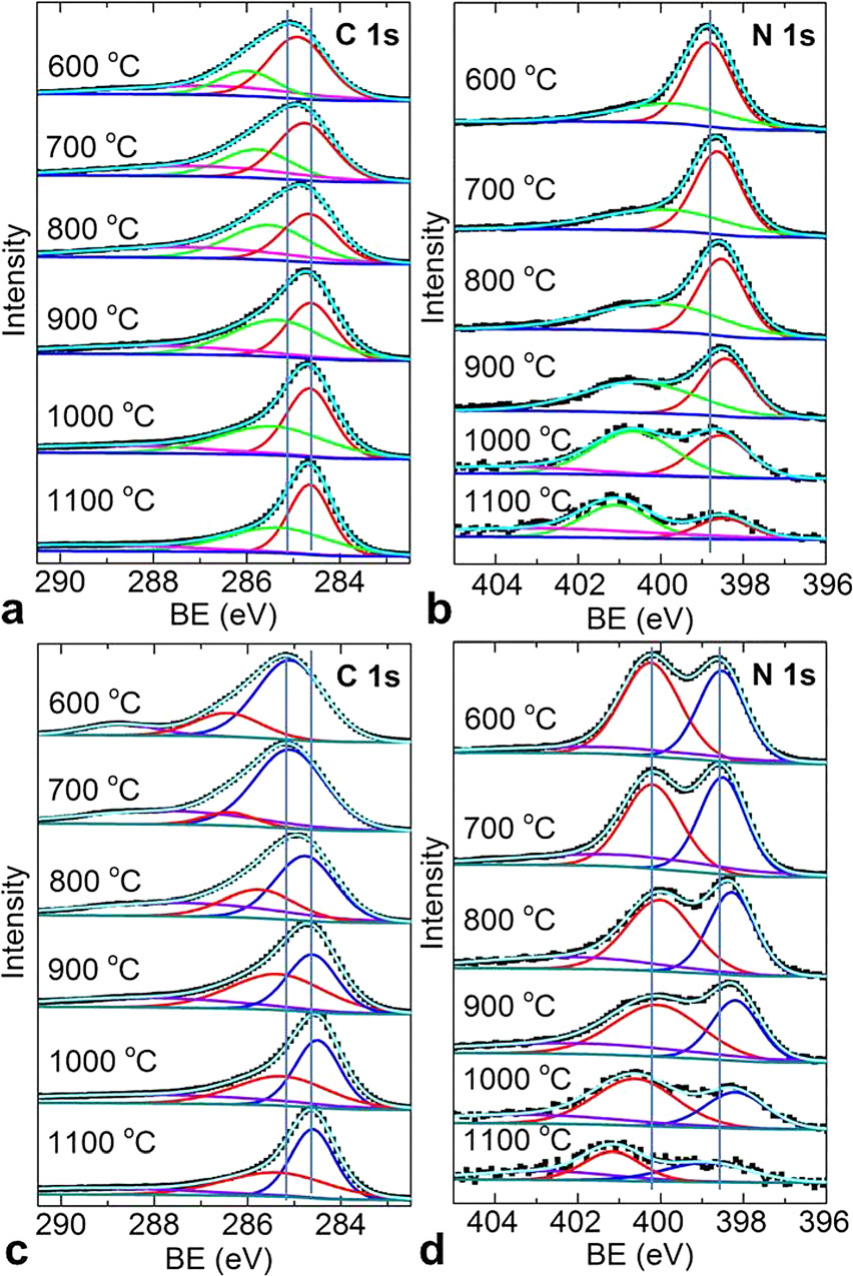
XPS spectra of nZDCs: a, c) C 1s and b, d) N 1s core-level spectra: b, d) before and c, d) after acid treatment. The experimental data is shown by solid square symbols, and the fittings, background, peaks, and envelop are shown by smooth lines. The corresponding carbonization temperature is also shown on each plot. The vertical guide lines represent the shifts in the main peak positions with carbonization temperature.

To understand further the carbon structures, the chemical natures of the C, N, and Zn atoms were determined by XPS elemental surveys. The comparative individual C 1s, N 1s, O 1s, and Zn 2p core-level spectra of all of the samples are shown in Figures 6, S21, and S22. For clarity, the spectra of the as-synthesized and acid-treated samples are grouped separately. In the ZIF-8 structure, the Zn=N4 coordination and the free rotational =CH_3_ groups on the imidazolate ring are the weakest possible links before ring decomposition,[10a] as is evidenced clearly by the structural collapse identified by the PXRD patterns and the early methyl signal in the TG–MS spectra. Thus, the increasing carbonization temperature results in a variation of the chemical interactions between the C, N, and Zn atoms, which is demonstrated by the clear qualitative differences with peak shifts and narrowing or broadening in the C 1s and N 1s spectra. The exact nature of the interactions is identified through the deconvolution and fitting of C 1s and N 1s peaks into multiple peaks at specific binding energies (BEs). A gradual narrowing and shift of the C 1s peak from a BE of 285.2 eV, which corresponds to sp^2^ C atoms bound to imidazole N atoms, to 284.6 eV for pure graphitic sp^2^ C phase with increasing carbonization (600 to 1100 °C) suggests that there is a considerable loss of N.[13a,^16^] The minor peaks situated at a BE of approximately 287 eV for the as-synthesized samples are attributed mainly to the N=C and/or Zn=C interactions, whereas the other peak at approximately 290 eV, which is more prominent in the spectra of the acid-treated samples (600 to 800 °C), is assigned to the C=O bonds.[13,16] The C 1s peak of ZIF-8 represents the coexistence of both the sp^2^ C atoms of the ring and the sp^3^ C atoms of the =CH_3_ groups. Similarly, the N 1s peak is deconvoluted to two prominent peaks. The main peak situated between BEs of 398.4 and 398.8 eV for all of the samples is assigned to pyridinic nitrogen atoms.[13,16,17] The second peak between BEs of 400.1 and 400.6 eV is assigned to pyrrolic nitrogen atoms, and a small shoulder above 401 eV is assigned to quaternary and oxidized nitrogen atoms (≥900 °C). A very weak peak for pyrrolic nitrogen atoms in the as-synthesized samples compared to the much more intense peak for the acid-treated samples, especially that carbonized at ≤800 °C, suggests that considerable Zn–N coordination remains. It is worth noting that the initial ZIF-8 shows a narrow symmetric peak at a BE of 399.4 eV for one form of nitrogen in the framework (Figure S21). The shift of the peak to higher BE is observed for metal-bound nitrogen atoms and is estimated to be up to 1 eV with respect to the peak for the pyridinic N atoms of the imidazole structure (398.8 eV).[18] This is well supported by a peak shift to a BE of 398.8 eV for a decomposed/carbonized sample at 600 °C. The increased carbonization temperature also results in pyridinic to pyrrolic and quaternary/oxidized transitions for the nitrogen atoms.[13,16,17]

The O 1s peak for the adsorbed atmospheric oxygen gives further interesting information on the (defective) coordination of the N and C atoms with the Zn atoms. Simple surface-adsorbed oxygen without strong chemical interactions shows a symmetric peak at a BE of 532 eV (e.g., see ZIF-8). However, the carbon samples always show broad two-peak behavior. A peak at a BE of 531.6 eV for the as-synthesized samples suggests a strong coordination with the Zn atoms without the formation of ZnO, which should show a peak at a BE of 531.1 eV.[2a] In addition to the adsorbed O (BE=532 eV), a promising peak at a higher BE of 533.4 eV clearly suggests C=O bonding owing primarily to the defective C, which is more clear in the samples after acid treatment (also see the C 1s peak at BE=289 eV).[13a,^16^] The Zn 2p core-level spectra of these ZIF-8-derived carbons also show interesting results for the residual Zn compared with those of other MOF carbons derived from carboxylate-bridged Zn atoms (MOF-5 and MOF-74).[2a] The formation of highly crystalline ZnO is seen if MOF-5 and MOF-74 are carbonized at 600 and below 900 °C, whereas completely Zn-free carbons are obtained at ≥900 °C. This is attributed to the reduction of ZnO with C to produce Zn vapor and CO_2_.[2a] However, the presence of Zn in ZIF-8 carbons with similar carbonization conditions (≥900 °C) can be attributed to the very strong Zn–N interactions. Note that no formation of zinc nitride adducts is seen.

**Figure 7:**
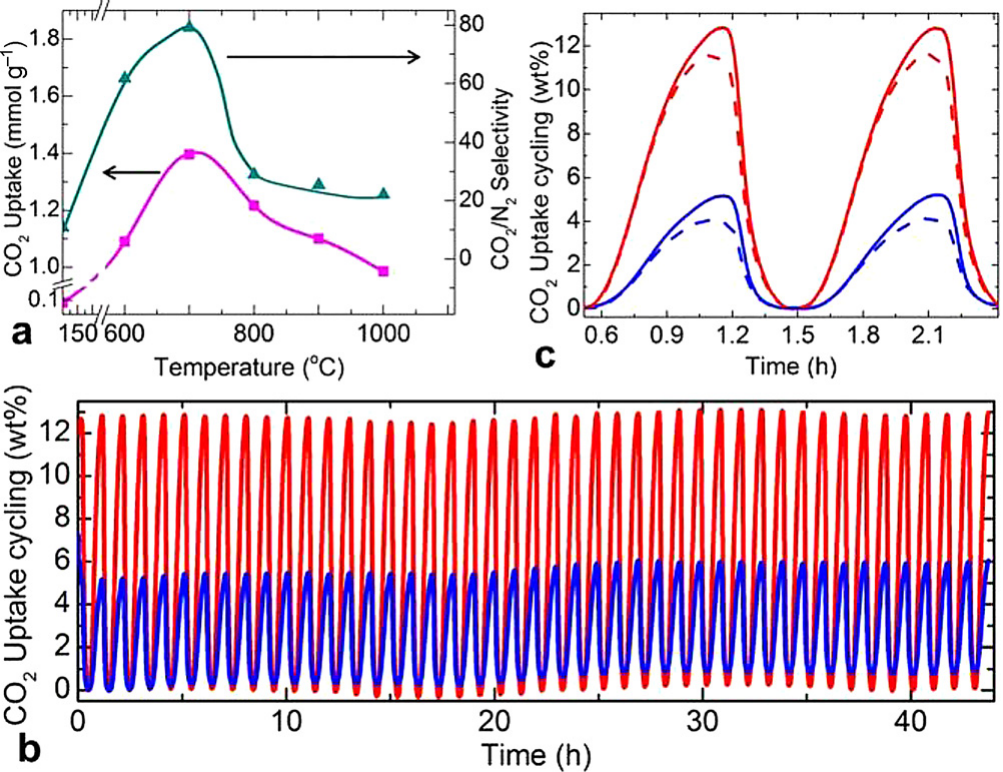
a) 25 °C CO_2_ uptake (at 0.15 bar, taken from isotherms in Figure 5) and calculated IAST CO_2_/N_2_ selectivity with respect to the carbonization temperature. Temperature-swing CO_2_ (100 % in red, 15 % balanced with 85 % N_2_ in blue) uptake cycling runs for b) 44 cycles and c) 2 cycles of nZDC700 (solid line) and nZDC1000 (dashed line). All cycling runs were measured by TGA under a continuous flow of the test gas at 1 bar and a swing temperature between 31 and 200 °C.

As shown in Figures 2 d and 7 a, the high pyrrolic N content obtained at low carbonization temperatures coincides with the high CO_2_ uptake tendency as well as the high CO_2_/N_2_ selectivity. The temperature-swing CO_2_ adsorption cycling tests between 31 and 200 °C under flowing CO_2_ at approximately 1 bar show very stable uptakes for several cycles (Figure 7 b and c, Figures S23 and S24). In agreement with the volumetric uptake isotherms, the N functionalities result in a clear advantage of nZDC700 over nZDC1000 for both the 100 % CO_2_ and 15 % CO_2_ (in 85 % N_2_) environments; the latter corresponds to the CO_2_ content in a flue-gas environment. These 4–5 wt % gravimetric uptakes of gas mixtures are in close agreement with the pure single-component volumetric uptakes at 0.15 bar. From Figures S23 and 24 as well as Table S3, it is also worth noting that the CO_2_ uptake is higher for ZDC700 than for ZDC1000 over the wide temperature range of 0–100 °C.

**Figure 8:**
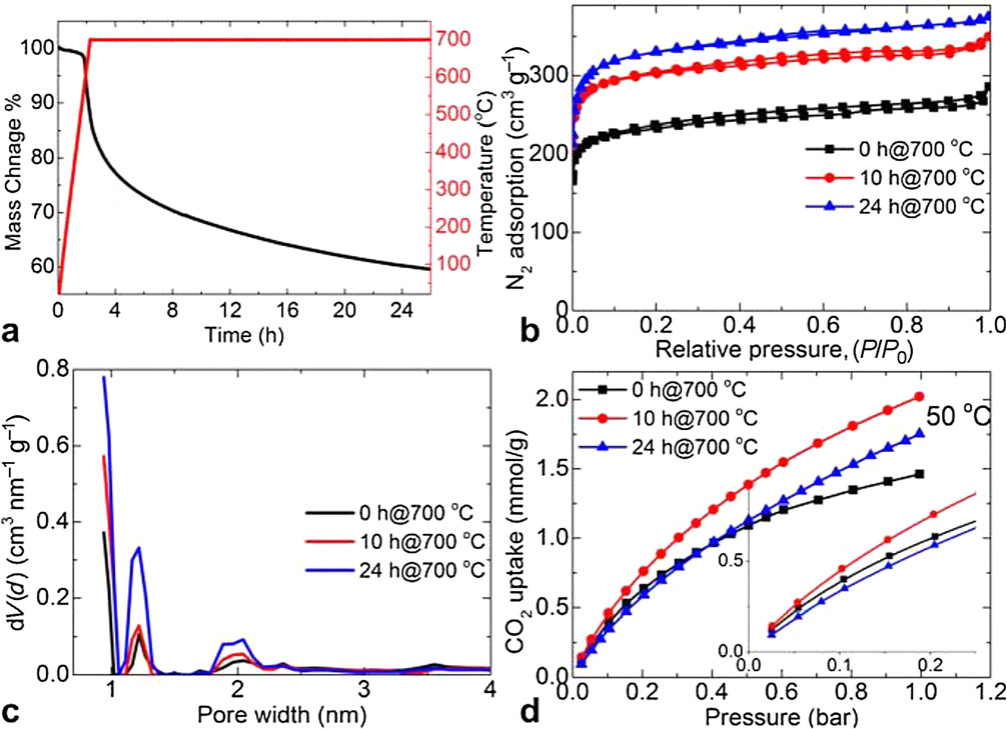
Influence of carbonization residence time: a) 700 °C isothermal TGA plot of mZIF-8 for 24 h, b) 77 K N_2_ adsorption–desorption isotherms of mZDC700 with carbonization residence times of 0, 10, and 24 h, and the corresponding c) pore size distribution plots and d) representative CO_2_ uptake isotherms at 50 °C. The inset shows the very-low-pressure CO_2_ uptake data at 50 °C.

Furthermore, the data shown in Figures 8 and S25–S27 give further structural insights into the effects of the heating rate (2–10 °C min^−1^) and carbonization residence time (up to 24 h). For example, the TGA for the prolonged carbonization of mZIF-8 at 700 °C shows a continuous sample mass loss up to 40 wt % at 24 h. Clearly, increasing the carbonization time from 0 to 24 h results in a considerable enhancement in porosity (e.g., the BET specific surface area increases from 915 to 1285 m^2^ g^−1^; Figure 8 b and c and Figure S25), in good agreement with the TGA mass loss and XPS elemental analysis (Figure S26), which suggest that ligand decomposition creates a more-porous carbon network. However, as shown in Figures 8 d and S25 the highest CO_2_ uptakes at 0, 25, and 50 °C under 1 bar are measured for a sample carbonized for 10 h rather than the samples carbonized for 0 or 24 h, which have the highest N content and porosity, respectively. Thus, the carbonization period is clearly one of the important parameters for obtaining an optimum carbon structure with high CO_2_ uptake. Similarly, the influence of heating rate on the porosity of carbonized samples from a new batch of ZIF-8 precursor is shown in Figure S27. The data represents all of the samples carbonized at 1000 °C for 6 h but with different heating rates. Among those, the rapid heating rate of 10 °C min^−1^ results in a highly reduced porous carbon, which could be caused by the explosive nature of sample decomposition to leave a more-collapsed network. The prolonged carbonization time of 24 h at this temperature did not produce any apparent changes to the specific surface area or pore volume with respect to those of the sample carbonized for 6 h.

**Figure 9:**
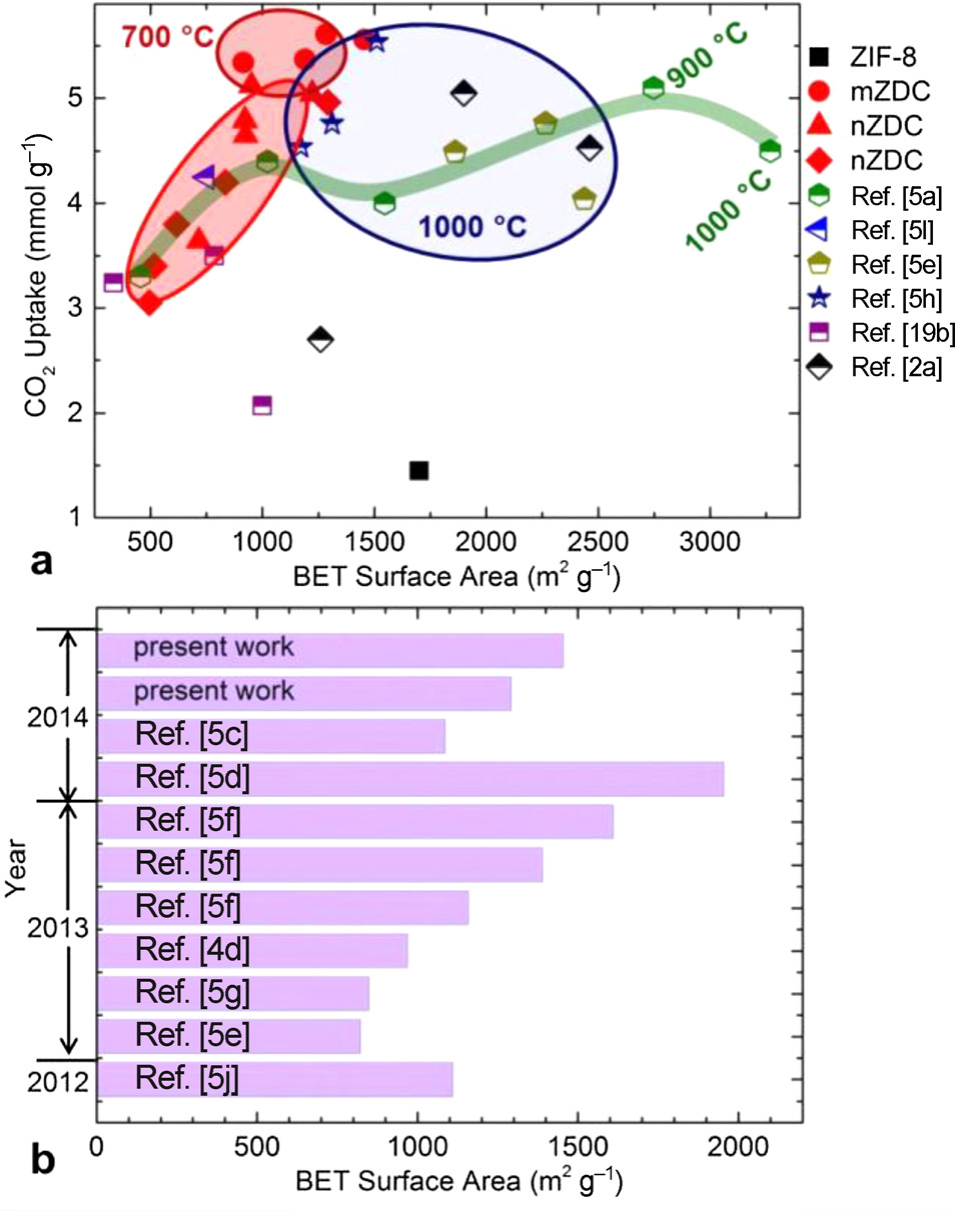
a) Comparative CO_2_ uptakes of MOF-derived carbons against BET specific surface area. As highlighted by the red circles, the ZDCs show far better CO_2_ uptakes, especially the mZDCs carbonized at 700 °C, compared with those of other functionalized[5a,l,17b] and KOH-activated^[5e, 5f]^ ZIF carbons and MOF-5, MOF-74, and MIL-53(Al) derived carbons.[2a] The data under the region with the dark blue circle is from the samples carbonized at 1000 °C. b) A comparison of the BET specific surface areas of direct carbonized ZIF-8 at 1000 (present work and Refs. [5c,e,g,j]), 900,[4d] and 800 °C.[5d,f]

The important porosities, BET specific surface areas, and CO_2_ uptake data at 0 and 25 °C under 1 bar for our ZDCs and previously reported MDCs (including N-free MOF-5, MOF-74, MIL-53, and N-containing ZIF carbons with and without a secondary carbon or functional source) are summarized in Figures 9 and S28. Compared with the MDCs with a carbonization temperature of 1000 °C, the ZDCs formed at 700 °C show the better CO_2_ uptakes (Table S3). For example, the uptake of 5–5.6 mmol g^−1^ at 0 °C and 1 bar for our n/mZDC700 with a specific surface area of 915–1285 m^2^ g^−1^ is much higher than or comparable with the literature values of 4 or 4.5–5.5 mmol g^−1^ for ZIF-8 carbonized at 1000 °C followed by KOH activation at 750 °C with a high specific surface area of 2437 m^2^ g^−1^ or FA-impregnated isoreticular ZIFs (IRZIFs; ZIF-8, -68, -69, -70) carbonized at 1000 °C with surface areas of up to 3200 m^2^ g^−1^.[5a,e,h] Also note the high CO_2_ uptake values of 1.5–2.4 mmol g^−1^ at pre- and postcombustion CO_2_ capture temperatures of 40–75 °C. The CO_2_ uptake of nZDC700 of approximately 2.4 mmol g^−1^ is more than twice the capacity of recent KOH-activated polymer-derived S-/N-doped carbons (1.0 mmol g^−1^) at 50 °C and 1 bar[19] and also higher those of other activated carbons with very high surface areas.[2a,14c] From the comparative data, we also note a wide range of BET specific surface areas for directly carbonized ZIF-8 (Figure 9 b). We attribute this discrepancy largely to the structural improvement of the ZIF-8 precursor. Even though MOFs represent well-defined pore structures, a range of surface areas have been reported, for example, 1000 to 2000 m^2^ g^−1^ for ZIF-8. It is also evident that samples of small crystals of ZIF-8 always show a lower surface area than those with large crystals;[5f] this could be due to an increase in structural defects or incomplete structure growth. The carbon derived from large crystals also shows a more-ordered in-plane structure [see high intensity (100) or (101) peaks in Figure S11 d] than the more turbostratic nature of nZDC. Hence, the larger-crystal-derived carbons show a more-open and ordered pore structure.

Finally, the comparative CO_2_ uptake isotherms of N-containing and N-free micro- and mesoporous MDCs with similar surface area and those of precursor ZIF-8 up to 1 bar (Figures S29 and S30) show the very clear advantage of ZDC700, which has a highly enhanced CO_2_ uptake at relatively low partial pressures. This direct comparison also ensures the importance of designing and fabricating functionalized microporous carbons as effective adsorbents for flue-gas CO_2_ capture. We also note that the CO_2_ uptake and the CO_2_/N_2_ selectivity of our nZDC700 are higher than the recently reported values for carbon derived from FA-ZIF-8 under similar conditions at 700 °C.[5a] Importantly, the ZIF-8 sample carbonized at 700 °C without acid treatment shows a significant advantage over ZIF-8 in terms of CO_2_ binding energy (>40 to ≈17 kJ mol^−1^), uptake (an order of magnitude greater at 0.15 bar and three times higher at 1 bar), and selectivity (three times higher; Table 1) for a sample mass loss of only approximately 30 wt % (by TGA, indicating a high yield). From this study, it can be inferred that it is important to perform a careful characterization of the dependence of CO_2_ uptake and selectivity on the carbonization temperature to identify the best conditions for each purpose.

## Conclusions

As demonstrated above through the complementary characterization techniques, MOF-derived carbon structures can be well optimized by simply controlling the carbonization conditions. Importantly, our in-depth investigation suggests that highly active and functional carbons can be produced efficiently from ZIF-8 by carbonization at approximately 700 °C, much lower than the often-reported carbonization temperature of 1000 °C and with much higher yield. The high-temperature carbonization can enhance the porosity but only at the expense of considerable loss of sample mass and also functional N sites. Thus, by controlling the carbonization temperature of ZIF-8 between 600 and 1000 °C, we show very different porous carbons with varied activated C and N sites. We also show a very slow evaporation rate of residual Zn metal in the structures carbonized at ≥900 °C, in contrast to the Zn-free carbons produced readily from MOF-5 and MOF-74. In the as-synthesized carbons, the porosity development with increasing carbonization temperature is correlated directly to the Zn metal pore decoration (at ≤800 °C), subsequent ligand decomposition, and Zn evaporation (≥900 °C). In addition to the usual porosity-governed CO_2_ uptake at high-pressure, a clear N functionality- and Zn-metal-assisted enhancement at low-pressure uptake is observed. For example, on the basis of their BET specific surface areas or micropore volumes, the Zn-containing carbons constantly show enhanced CO_2_ uptake. After acid treatment, the samples with the same porosity and a high N content, mainly of the pyrrolic type, show highly enhanced CO_2_ uptakes at very low partial pressures; thus, these samples also show a high CO_2_/N_2_ selectivity up to three times greater than that of the sample carbonized at 1000 °C. Furthermore, a clear N- and Zn-metal-enhanced CO_2_ binding energy is observed. In summary, we have shown important structural insights into the ZIF-8-derived carbons. This approach facilitates the development of highly active and functionalized carbon structures.

## Experimental Section

### Synthesis

In a typical synthesis of ZIF-8 nanosized crystals (nZIF-8), a methanol solution (250 mL) of Zn(NO_3_)_2_**⋅**6 H_2_O (6.1467 g) was added slowly to a methanol solution (250 mL) of 2-methylimidazole (6.7873 g) with stirring (see Supporting Information) at room temperature. After a few minutes, a milky solution formed from the clear precursor solutions (Figure S1) and was then left to settle for 24 h. The top clear solution was decanted, and the white precipitate was collected by centrifugation with methanol washing. The microsized ZIF-8 crystals (mZIF-8) were synthesized in a similar way but with the addition of 1-methylimidazole as a moderator.[5g,7d] Both the as-synthesized ZIF-8 samples were later outgassed at 180 °C under dynamic vacuum for 24 h. MOF-5 was synthesized according to our earlier report.[2a]

In a typical carbonization process, ZIF-8 (≈500 mg) was placed in an alumina boat (1×1.5×5 cm) and then transferred into a horizontal tube furnace. The furnace tube was closed with a gas feed through the end seals, and the sample area was purged thoroughly with nitrogen. The nitrogen flow was maintained throughout the reaction. The carbonizations at 600–1100 °C were performed for 6 h at a given temperature with a heating rate of 5 °C min^−1^. The carbons obtained at different carbonization temperatures from nZIF-8 were named as nZDC*xxx*, (*xxx*=600, 700, 800, 900, 1000, and 1100 and represents the carbonization temperature from 600 to 1100 °C). All samples for further characterizations were handled in ambient air. The residual Zn adducts in the as-synthesized carbon samples were removed by treatment with HCl: the as-synthesized batch sample (100 mg) was added to HCl (33 %, 10 mL) in a 20 mL vial, and the mixture was stirred at room temperature for 24–72  h. The samples were then washed with deionized water and dried in a vacuum oven at 200 °C.

### Characterization

Combined TGA (Setsys analyzer from Setaram) and MS (OmniStar spectrometer from Pfeiffer Vacuum) at 600–1000 °C was performed with dry samples under an Ar flow with different heating rates of 2–10 °C min^−1^ and an isothermal step of 0–24 h. The TGA mass losses were recorded after background correction for the empty alumina crucible. Powder X-ray diffraction (Stoe Stadi-P diffractometer, CuK_α_ radiation) was performed by filling a 0.5 mm diameter glass capillary with the sample under ambient conditions. Raman spectroscopy (514.5 nm laser, Renishaw instrument) was performed with pressed powder samples on a glass slide. The XPS (AlK_α_ radiation, Thermo Scientific spectrometer), SEM (Jeol microscope), and TEM (Jeol microscope) were conducted with samples supported on carbon tape or a carbon-coated copper TEM grid. The porosity and gas adsorption–desorption isotherms up to 1 bar were determined with a Quantachrome Autosorb-iQC analyzer with samples cooled to 77 and 298 K with liquid nitrogen and a water bath, respectively. The specific surface areas were determined from the 77 K N_2_ desorption isotherm in a relative pressure range between 0.01 and 0.2, according to the BET method. The NLDFT method with slit/cylindrical pores was applied to the desorption isotherm (10^−2^ to 0.99, *P*/*P*_0_) to obtain the pore size distributions and cumulative pore volumes.[20] The high-pressure CO_2_ adsorption isotherms up to 30 bar and at 0–75 °C (maintained with an ice bath and a precisely controlled tube furnace) were measured with a PCTPro 2000 instrument. The isosteric heats of adsorption (*Q*_st_) were determined from the uptake isotherms measured at 0, 25, 50, and 75 °C by applying the Clausius–Clapeyron relation. The *Q*_st_ values were calculated point by point from the raw isotherm data without using any isotherm fit models. The samples were degassed at 150 °C overnight under dynamic vacuum before the gas adsorption measurements. The IAST (see Supporting Information) was applied to calculate the CO_2_/N_2_ selectivity *S*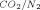. IAST predicts the mixture adsorption equilibriums from single-component adsorption isotherms through the relationship *S*_1/2_=(*q*_1_/*q*_2_)/(*p*_1_/*p*_2_); *q*_1_ and *q*_2_ are the CO_2_ and N_2_ uptake capacities in mmol g^−1^ at partial pressures of *p*_1_ (0.15 bar) and *p*_2_ (0.85 bar), respectively.
