# Supplementary material for: Tuning of ZIF-Derived Carbon with High Activity, Nitrogen Functionality, and Yield – A Case for Superior CO2 Capture
Source: ChemSusChem. 2015 Apr 27;8(12):2123–32. doi: 10.1002/cssc.201403402 (PMC4515097; doi:10.1002/cssc.201403402)
Supplement: Supplementary file 1 [file cssc0008-2123-sd1.pdf]

CHEMISTRY & SUSTAINABILITY
CHEMSUSCHEM
ENERGY & MATERIALS

Supporting Information

Tuning of ZIF-Derived Carbon with High Activity, Nitrogen Functionality, and Yield – A Case for Superior CO_2 Capture

Srinivas Gadipelli* and Zheng Xiao Guo*[a]

cssc_201403402_sm_miscellaneous_information.pdf

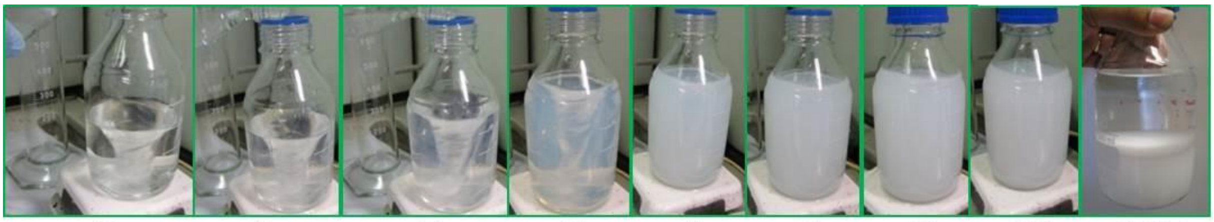

Figure S1. Showing the ZIF-8 synthesis method by room temperature magnetic stirring from methanol solutions of precursors; $Zn(NO_3)_2.6H_2O$ and 2-methylimidazole. $Zn(NO_3)_2.6H_2O$ dissolved in methanol was slowly added to 2-methylimidazole methanol solution while stirring. The numbers on the pictures are in minutes counted from the moment of adding solvents. The precipitation was later left overnight (O/N) for settling.

IAST (ideal adsorbed solution theory) is applied to calculate the CO_2/N_2 selectivity, $S_{CO2/N2}$. IAST predicts the mixture adsorption equilibriums using single-component adsorption isotherms and is defined as;

S (selectivity of CO_2/N_2) = $(q_1/q_2)/(p_1/p_2)$,

where q_1 and q_2 are the CO_2 and N_2 uptake capacities in mmol g^{-1} at partial pressures of p_1 (=0.15 bar) and p_2 (=0.85 bar), respectively

1. Doyun Lee, Chengyi Zhang, Chuan Wei, Brandon L. Ashfeld and Haifeng Gao, Hierarchically porous materials via assembly of nitrogen-rich polymer nanoparticles for efficient and selective CO_2 capture, J. Mater. Chem. A, 2013, 1, 14862.
2. Witold M. Bloch, Ravichandar Babarao, Matthew R. Hill, Christian J. Doonan, and Christopher J. Sumby, Post-synthetic Structural Processing in a Metal−Organic Framework Material as a Mechanism for Exceptional CO_2/N_2 Selectivity, J. Am. Chem. Soc. 2013, 135, 10441−1044.
3. Tae-Hyun Bae, Matthew R. Hudson, Jarad A. Mason, Wendy L. Queen, Justin J. Dutton, Kenji Sumida, Ken J. Micklash, Steven S. Kaye, Craig M. Brown and Jeffrey R. Long, Evaluation of cation-exchanged zeolite adsorbents for post-combustion carbon dioxide capture, Energy Environ. Sci., 2013, **6**, 128-138.

Heat of adsorption:

The high initial heat of adsorption is attributed to the interaction of CO_2 molecules with the strong binding energy sites, i.e., slit/cylindrical narrow size pores,[$] coordinatively unsaturated metal centres and functional nitrogen groups. Thus the reduction in interaction is expected with the filling pressure as the strong adsorptive sites are occupied. The increased isosteric heat of adsorption with further increase in pressure is due to the increased interactions between CO_2-porous support as well as CO_2-CO_2.

$ Normally the high heat of adsorption is expected when adsorbates form the one or two-layer films within the pores. When the pores are bigger the multi-layer adsorption is expected, however the interaction energies are expected to be very weak as third and higher number of layers is far from the adsorbent.

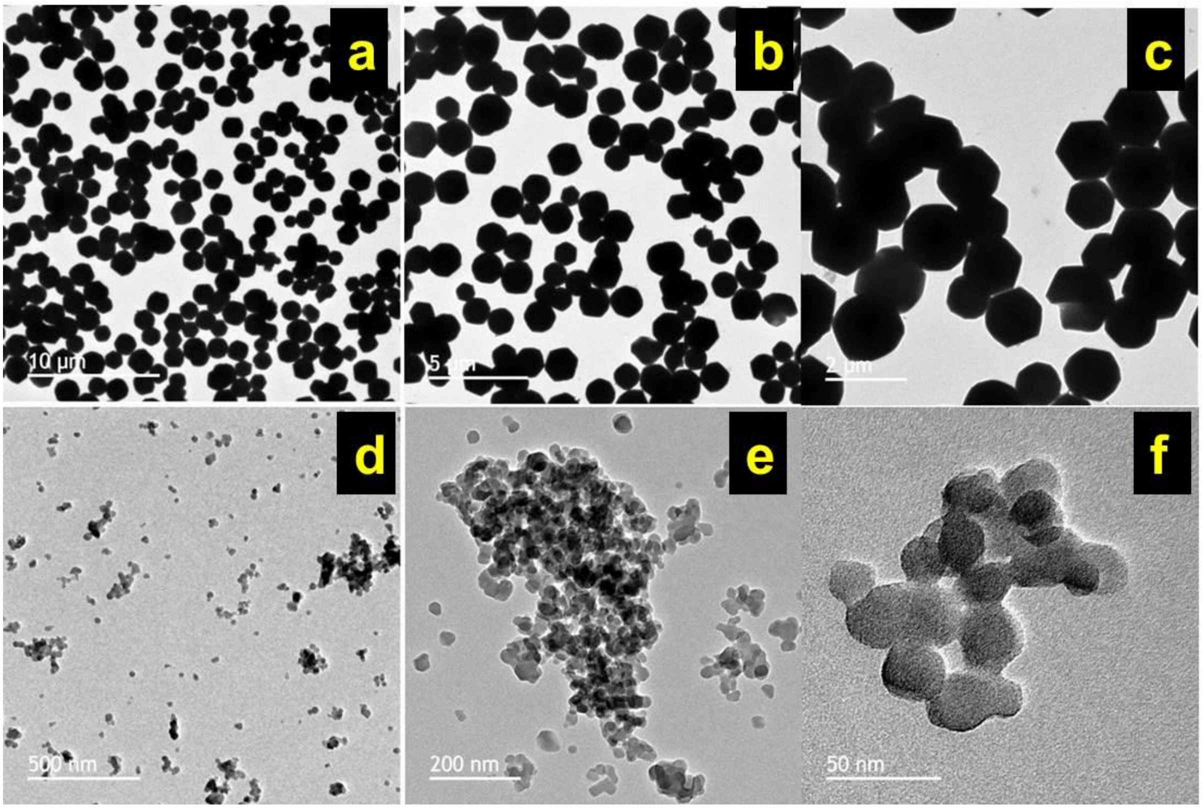

Figure S2. TEM images of ZIF-8 with different magnifications (a-c) microcrystals & (d-f) nanocrystals.

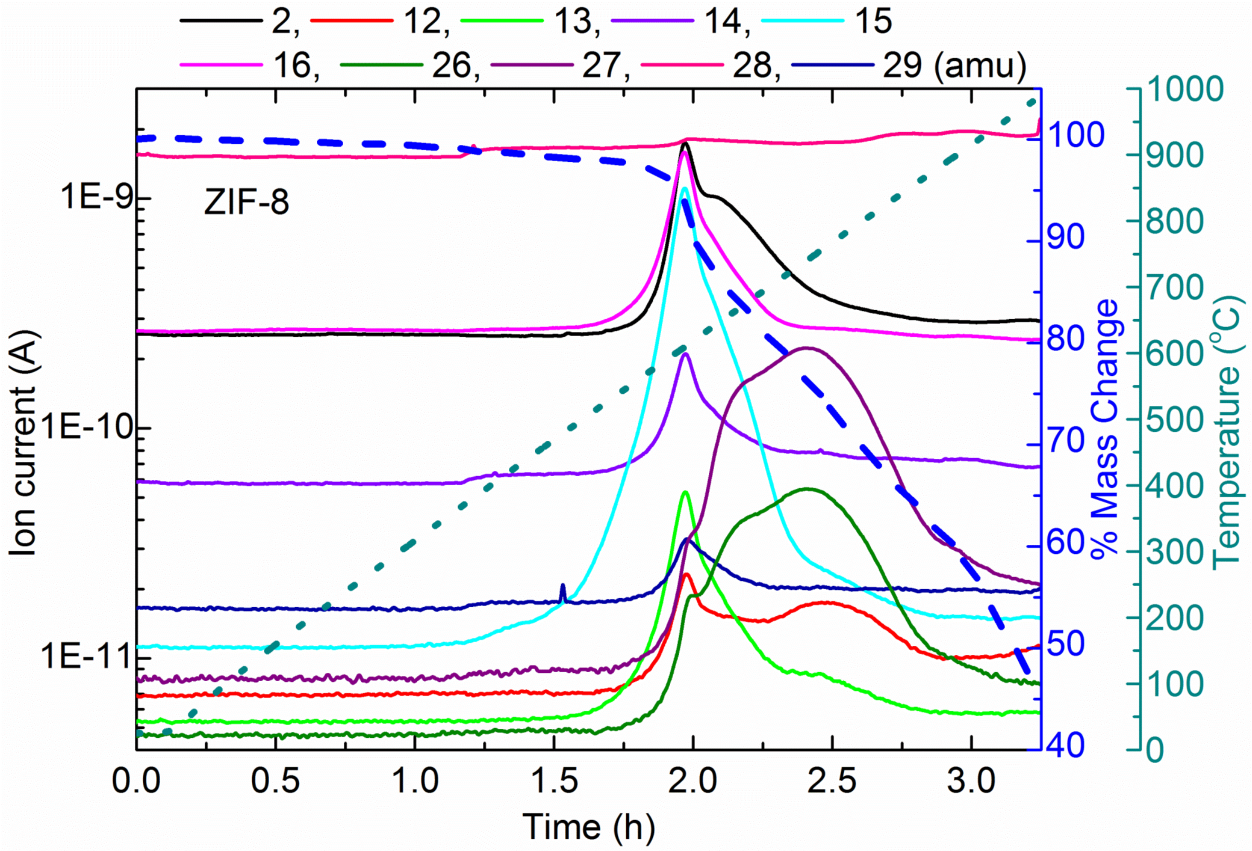

Figure S3. Simultaneous thermogravimetric and mass spectroscopy analysis of ZIF-8 reveals a framework decomposition/carbonization temperature ~600 °C, and evolution of various gaseous mixtures; H_2, C, C_xH_y, C_xN_y or both, $C_xN_yH_z$, identified by atomic mass unit (amu); 2-H_2, 12-C, 13-CH, 14-CH_2, 15-CH_3, 16-CH_4, 26-C_2H_2 or CN, 27-HCN, 28-H_2CN or N_2, 29-H_2CNH.

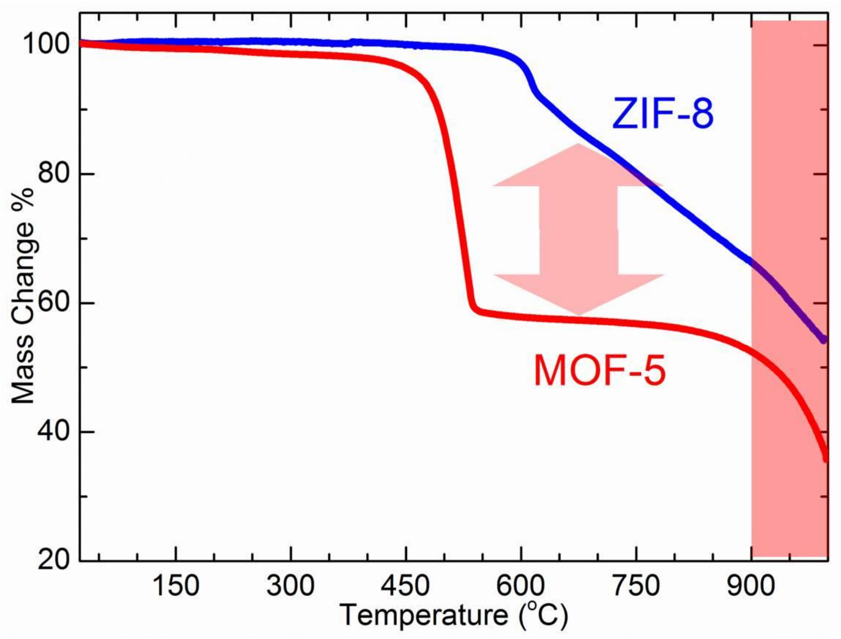

Figure S4a. TGA analysis of MOF-5 and ZIF-under Ar flow. The two-step sharp mass-loss in MOF-5 is attributed to the rapid decomposition of carboxylates at ~500 °C and Zn evaporation with further gasification of carbon above 900 °C due to the ZnO reduction with carbon as ZnO + C → Zn + CO(2).[2a]

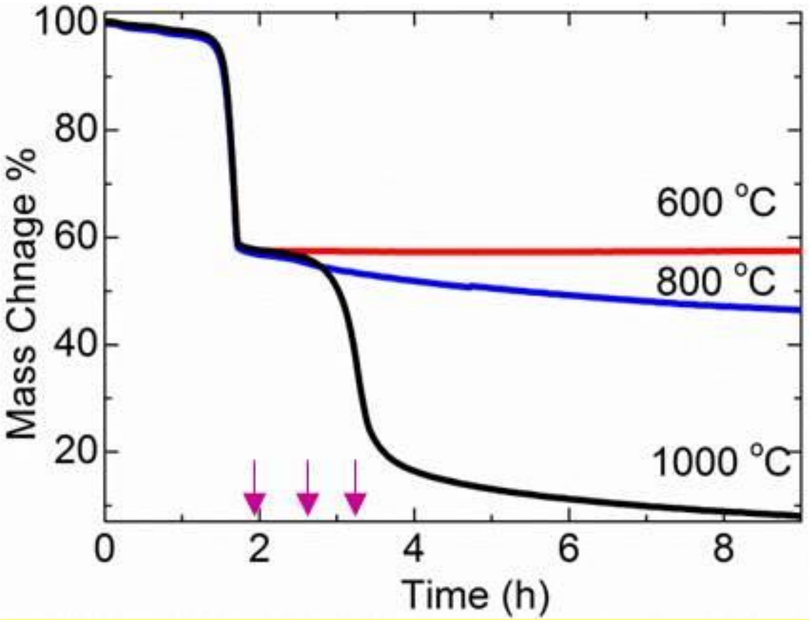

Figure S4b. TGA isothermal carbonization plotsMOF-5 at (600-1000) °C with residence time up to 7 h. The arrows represent the specific heating time (at 5 °C min^{-1}) to reach carbonization temperature.

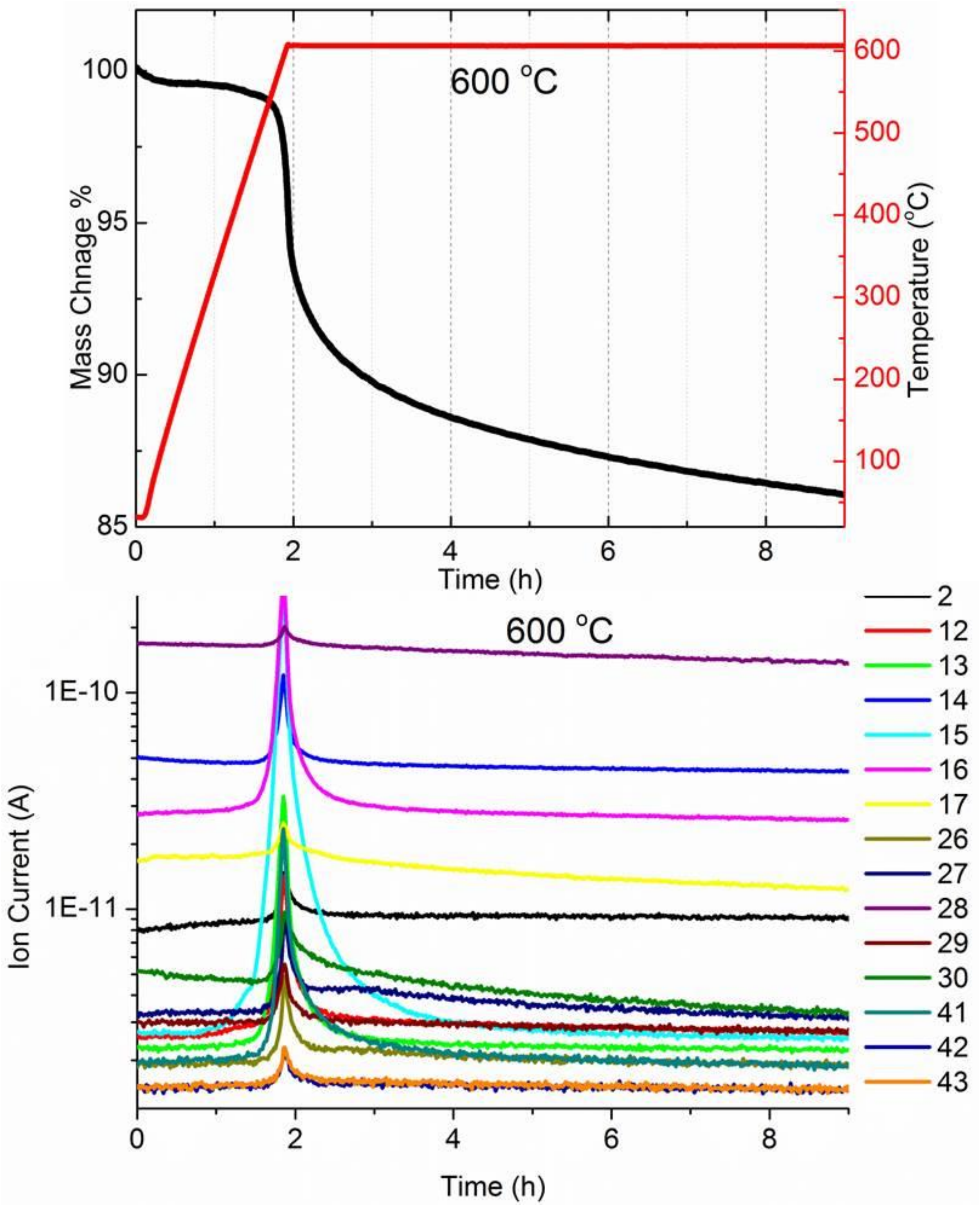

Figure S5. A combined TG-MS analysis of carbonization of ZIF-8 at 600 °C with heating rate of 5 °C and dwelling time of 7 h. In addition to mass-loss of ~5 wt% during heating to 600 °C a further gradual mass-loss of ~8 wt% is observed with isothermal step at 600 °C, which seems continuing for longer period. The MS amu signals show that this isothermal mass-loss is mainly due to continuous decomposition of methyl groups (amu of 15-CH_3, 13-CH, 30-C_2H_6 recombination of CH_3 radicals) on ligand.

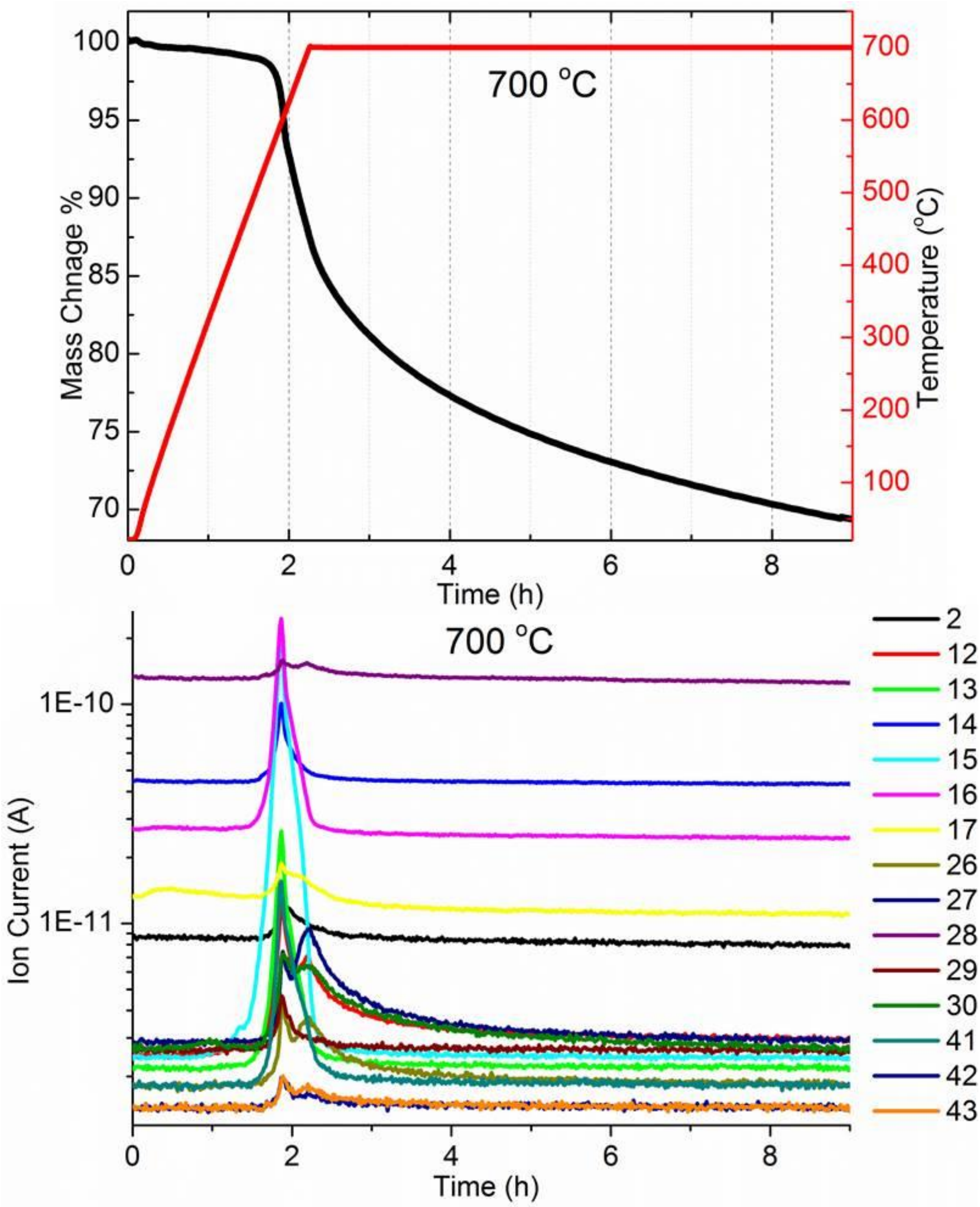

Figure S6. A combined TG-MS analysis of carbonization of ZIF-8 at 700 °C with heating rate of 5 °C and dwelling time of 7 h. In addition to mass-loss of ~15 wt% during heating to 700 °C a further rapid mass-loss of ~15 wt% is observed with isothermal step at 700 °C, which seems continuing for longer period. Unlike 600 °C, during the isothermal step the MS shows a clear additional signals related to the N-containing complexes at amu of 17-NH_3, 26-NC, 27-NCH, 28-N_2.

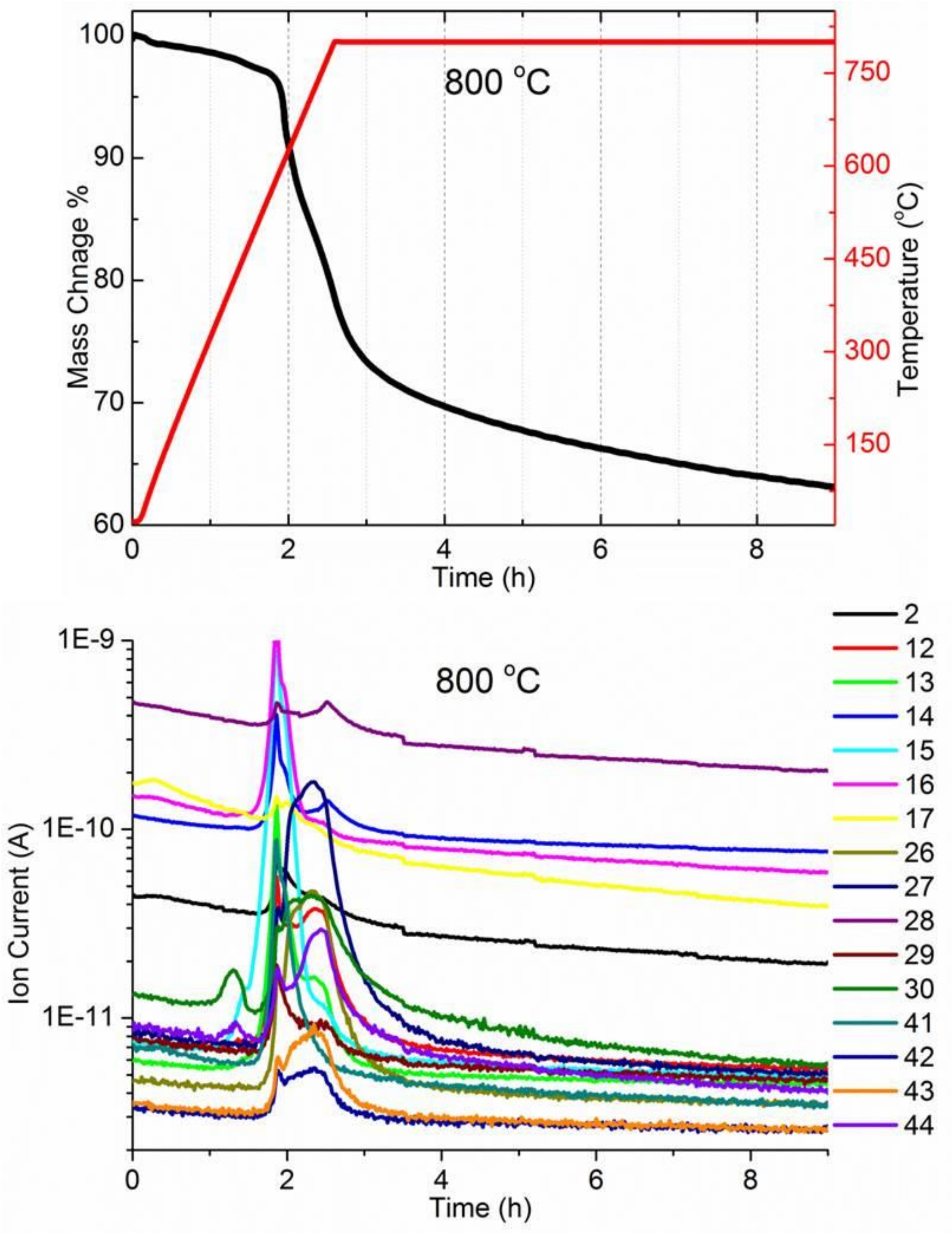

Figure S7. A combined TG-MS analysis of carbonization of ZIF-8 at 800 °C with heating rate of 5 °C and dwelling time of 7 h. In addition to mass-loss of ~22 wt% during heating to 800 °C a further mass-loss of ~16 wt% is observed with isothermal step at 800 °C, which seems continuing for longer period. A much more ligand decomposition is seen.

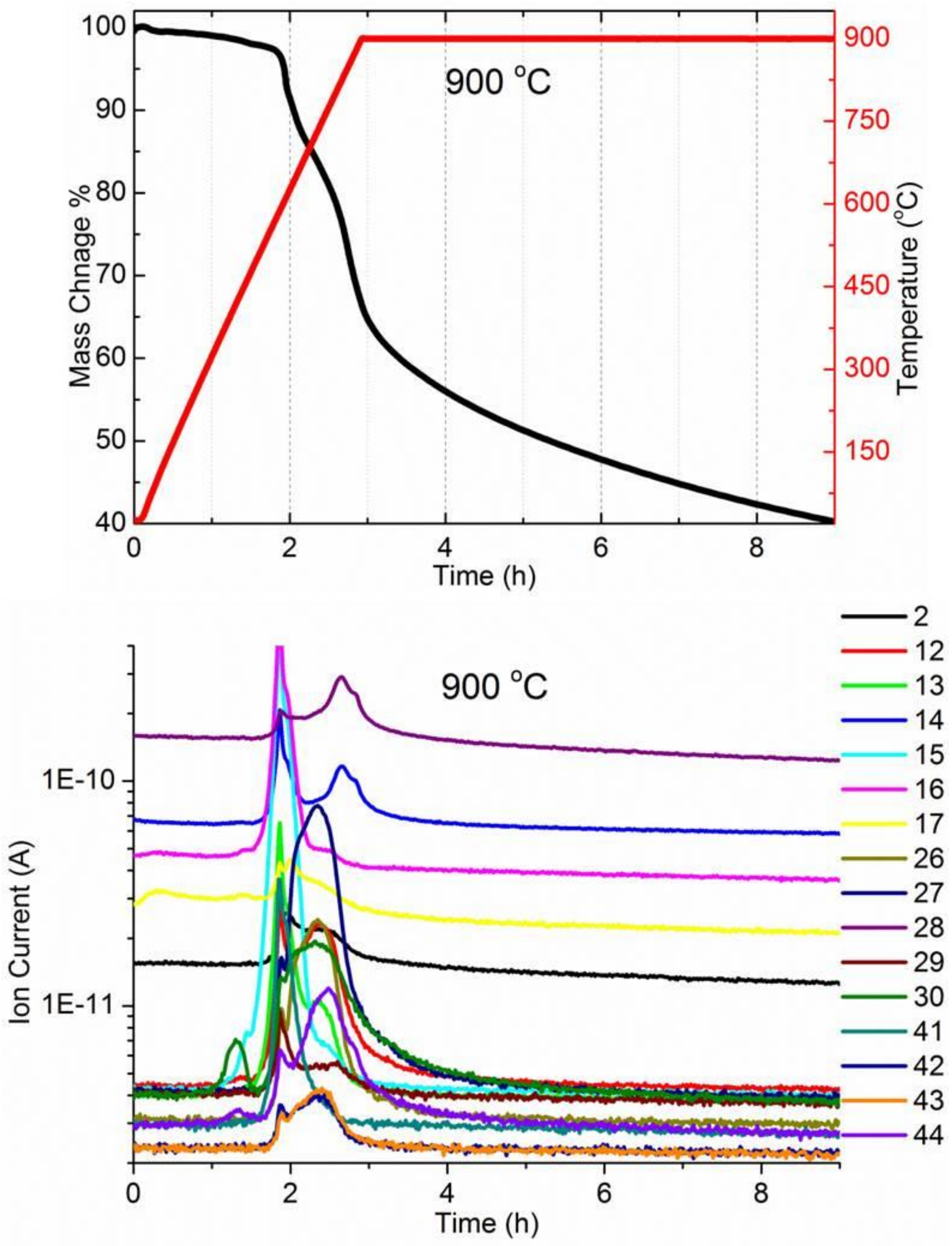

Figure S8. A combined TG-MS analysis of carbonization of ZIF-8 at 900 °C with heating rate of 5 °C and dwelling time of 6 h. A rapid mass-loss of up to 60 wt% is observed after 6 h carbonization. A clear and more intense N-& C-decomposition signals can be seen at amu of 12 (C), 14 (CH_2), 26 (NC), 27 (NCH), 28 (N_2).

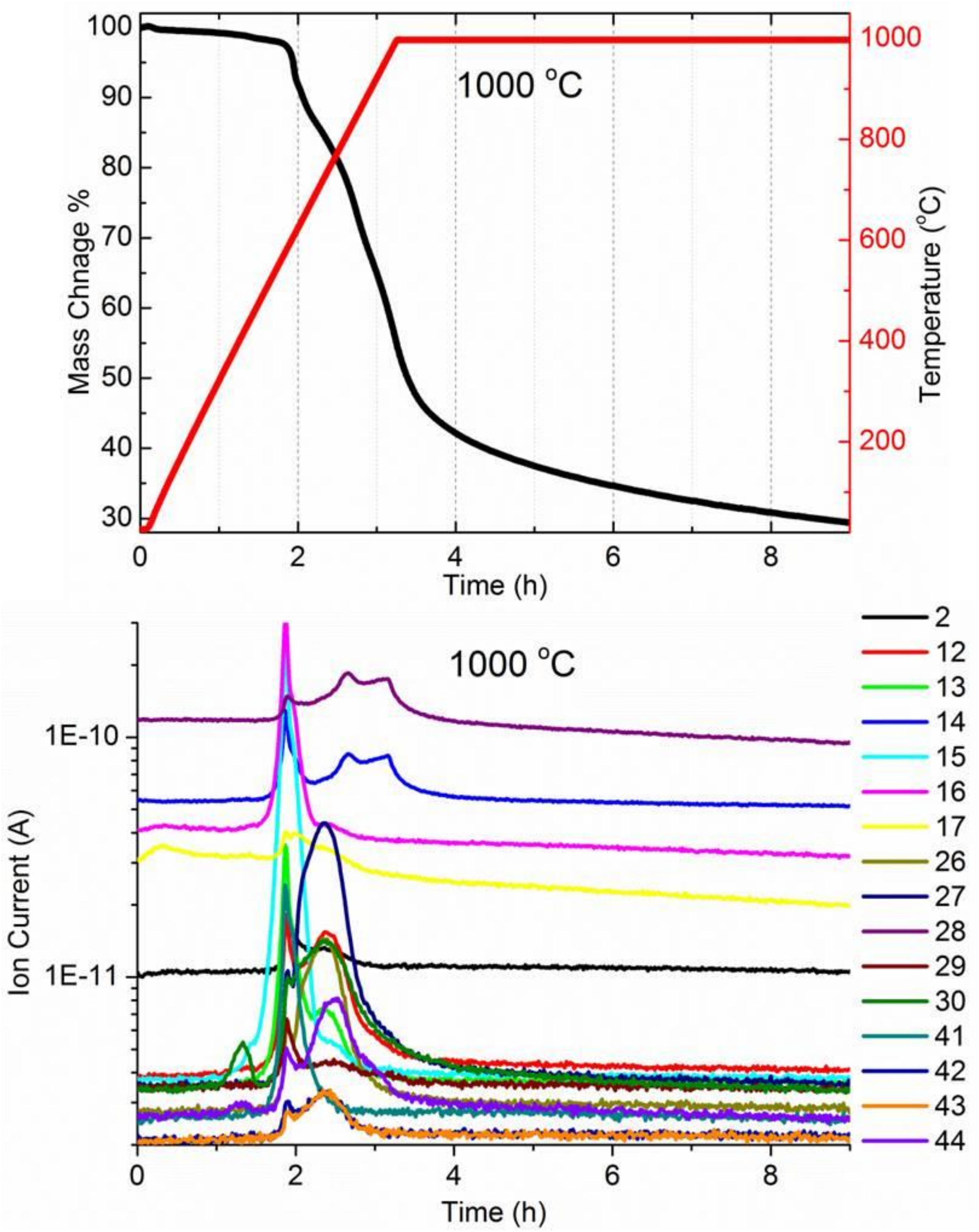

Figure S9. A combined TG-MS analysis of carbonization of ZIF-8 at 1000 °C with heating rate of 5 °C and dwelling time of 6 h. A rapid mass-loss of up to 70 wt% is observed after 6 h carbonization. A clear and more intense N-& C-decomposition signals can be seen at amu of 12 (C), 14 (CH_2), 26 (NC), 27 (NCH), 28 (N_2).

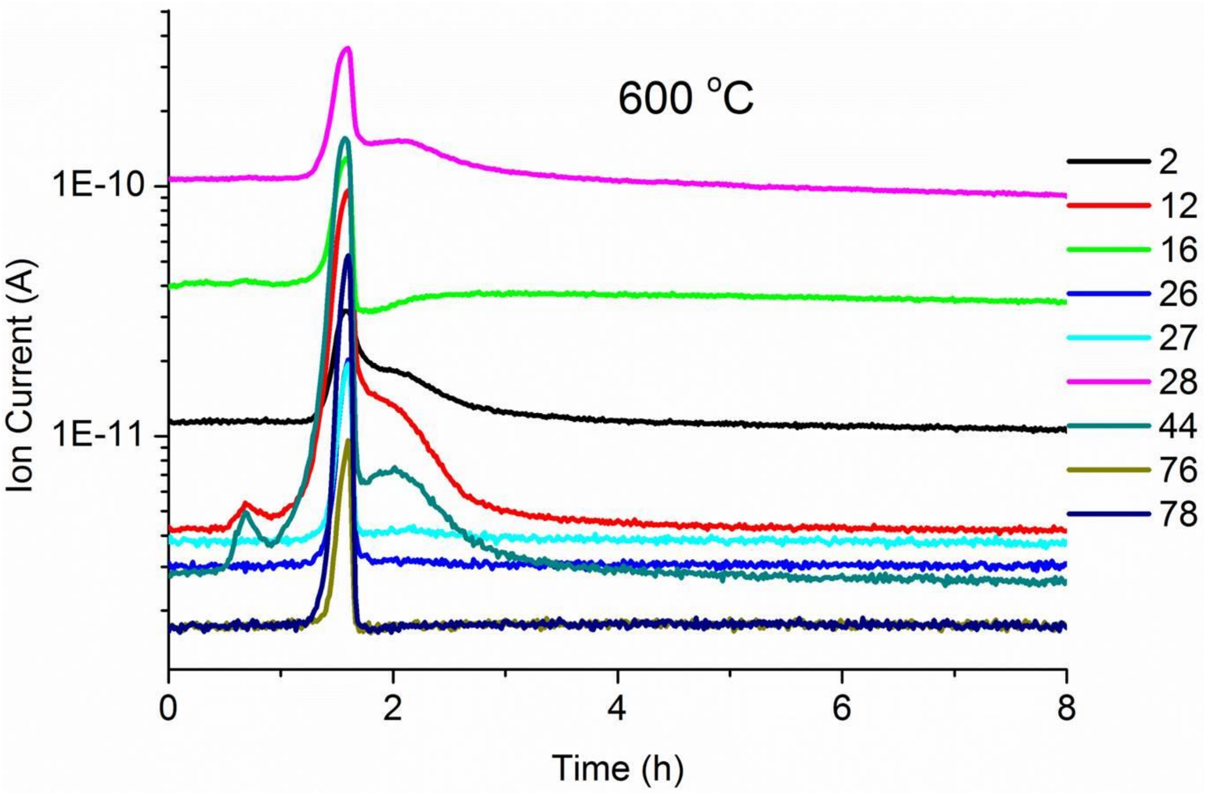

Figure S10. A combined TG-MS analysis of carbonization of MOF-5 at 600 °C with heating rate of 5 °C and dwelling time of 6 h. After rapid mass-loss of up to 40 wt% due to decomposition of carboxylates (see mass signals at 12 (C), 28 (CO), 44 (CO_2)) and ligand benzene ring (2 (H_2), 16 (CH_4), 26 (C_2H_2), 28 (C_2H_4), 76 & 78 (C_6H_6)) no further mass-loss is detected.

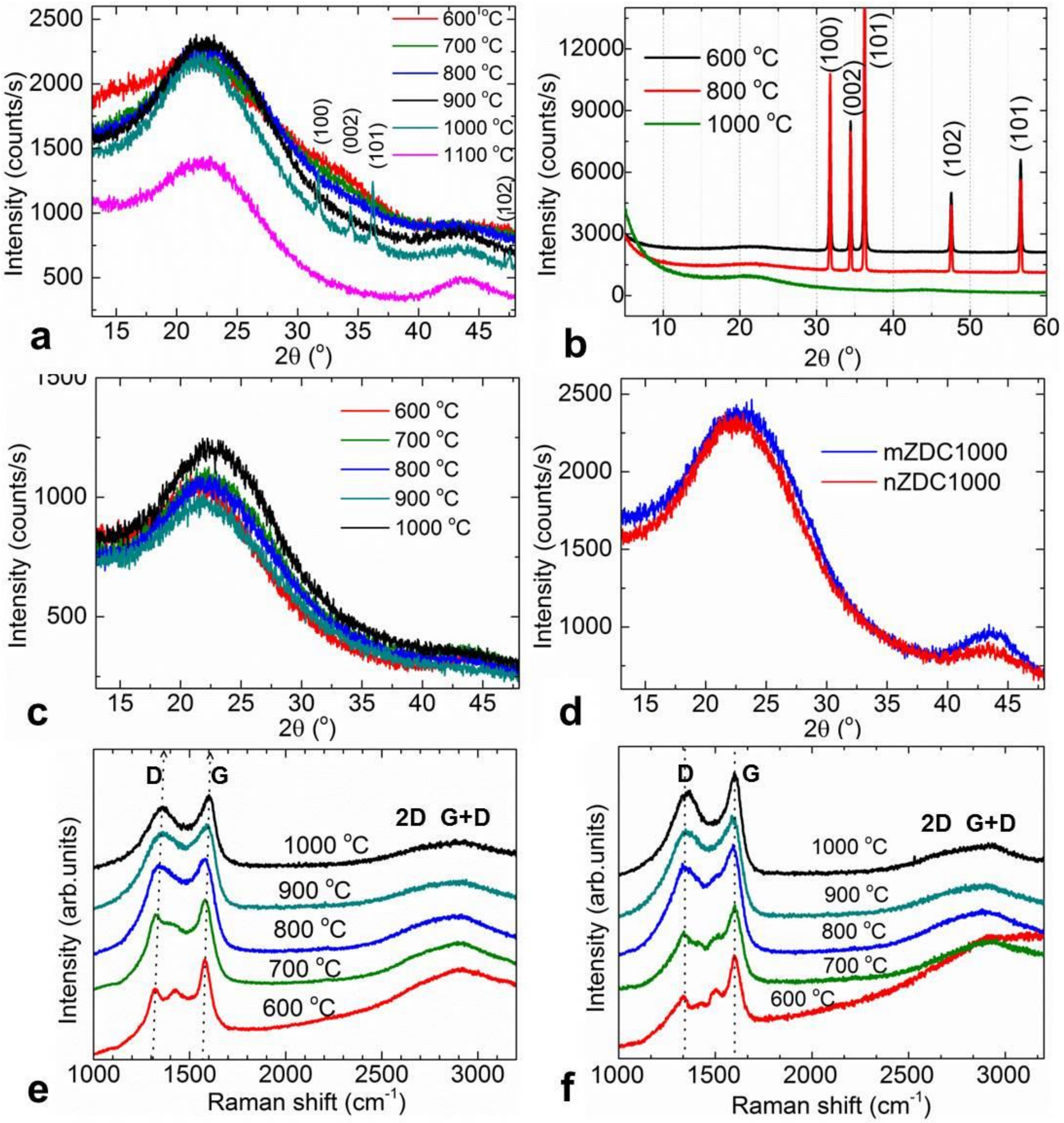

Figure S11 PXRD patterns (a-d) and Raman spectra (e-f) of the samples derived at various carbonization temperatures between 600-1000 °C. PXRD of as-synthesized carbons of ZIF-8 and MOF-5 are shown in (a) and (b) respectively. PXRD of acid-treated carbons of (a) are shown in (c) and (d) shows a comparative PXRD patterns of mZIF-8 and nZIF-8 derived carbons at carbonization temperature of 1000 °C. With increasing carbonization the growing peak intensity both at inter-layer (002) and in-plane (100) or (101) represents a more ordered graphitic structure, which is also evident from an inter-layer (002) peak shift from 21.8° to 22.6° of 2 theta. a) The 900 °C carbonized sample left in air for several days shows additional sharp diffraction peaks from the hexagonal ZnO similar to the MOF-5 carbons (b). d) A clear high intense (100) or (101) peak in the region around 44° of 2 theta represents more ordered in-plane structure in the mZIF-8 carbons. Similarly a small peak shift to higher angles in the region around 23° of 2 theta (~22.6° to ~23.2°) of mZIF-8 carbon also represent more graphitic order than nZIF-8 carbon, which is more turbostratic.

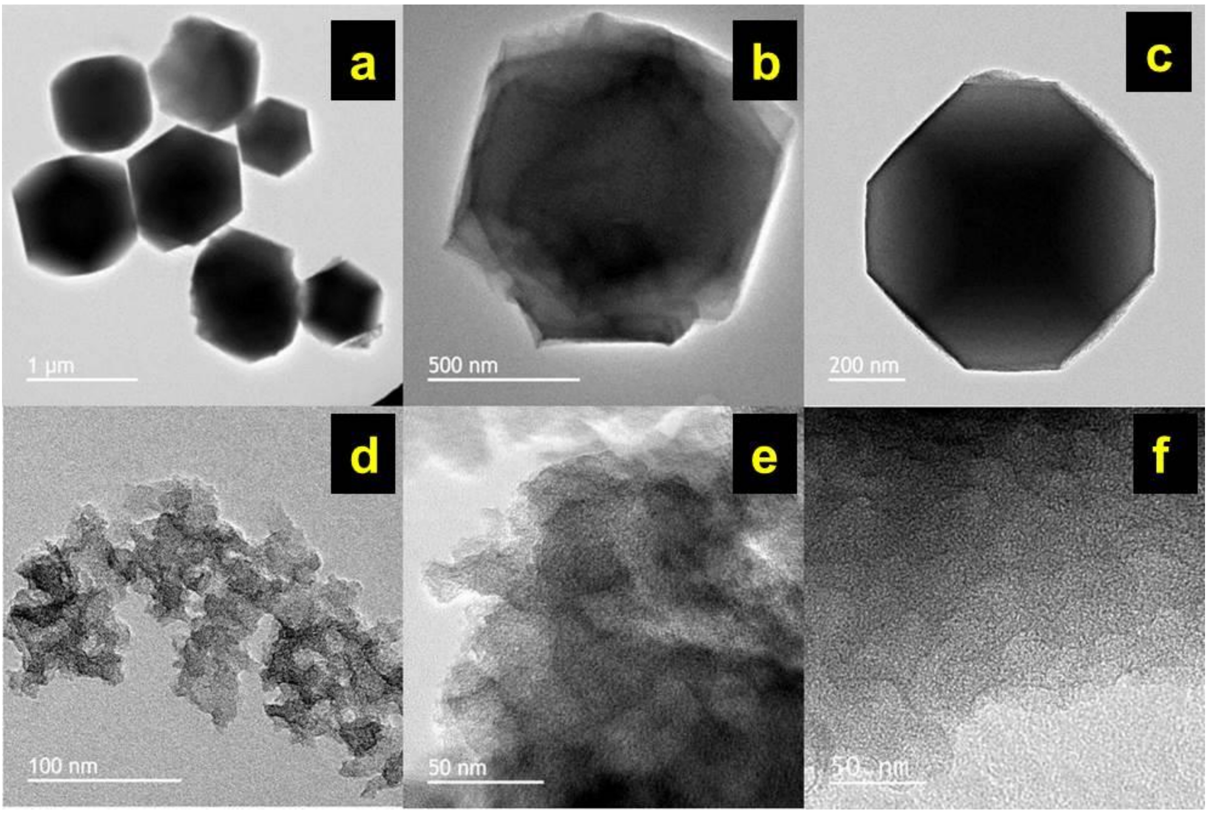

Figure S12. TEM images of ZDC1000 with different magnifications (a-c) microcrystals & (d-f) nanocrystals.

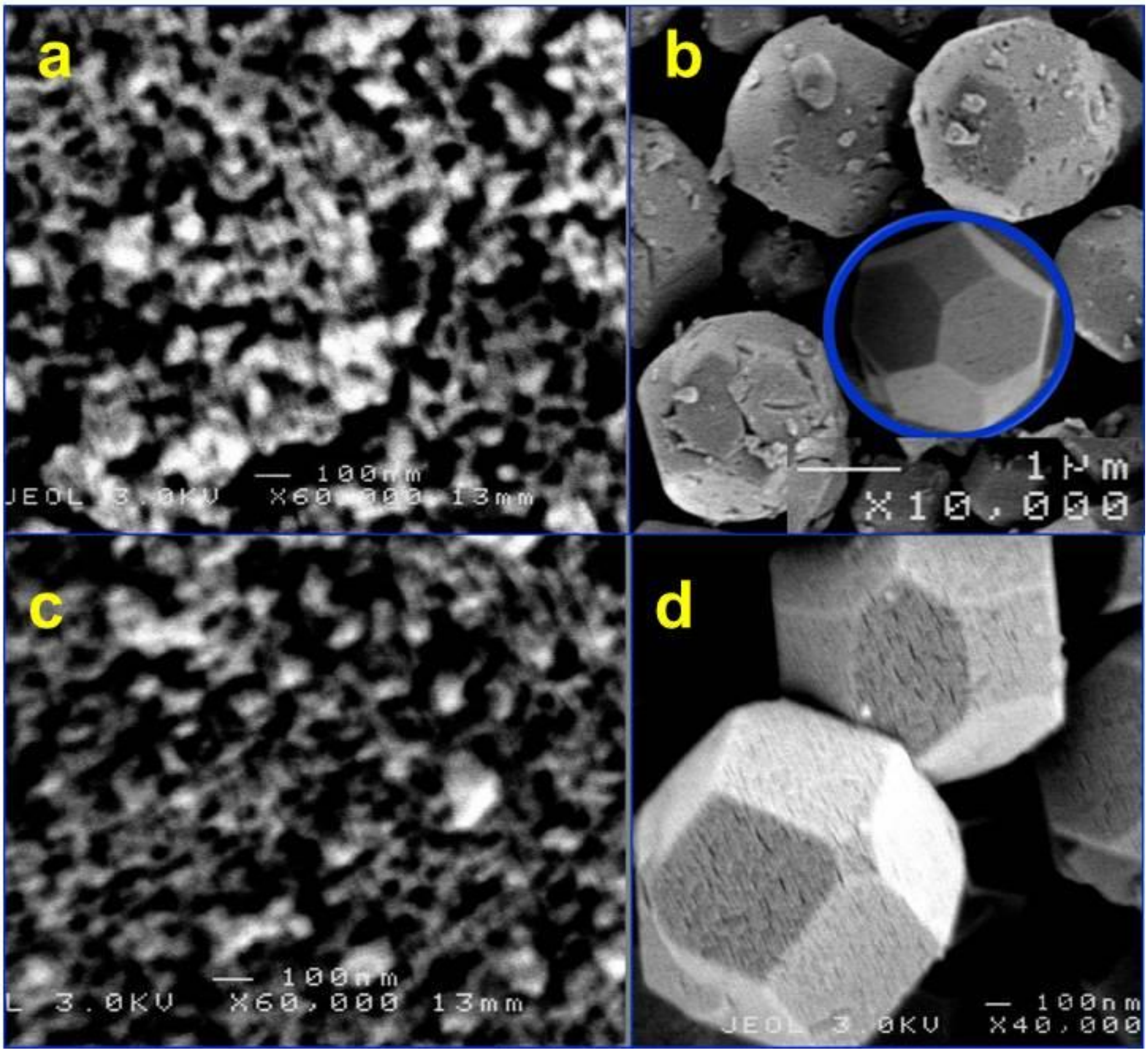

Figure S13. SEM images of ZIF-8 (a-b) and ZDC1000 (c-d). (a & c) nanocrystals & (b & d) microcrystals.

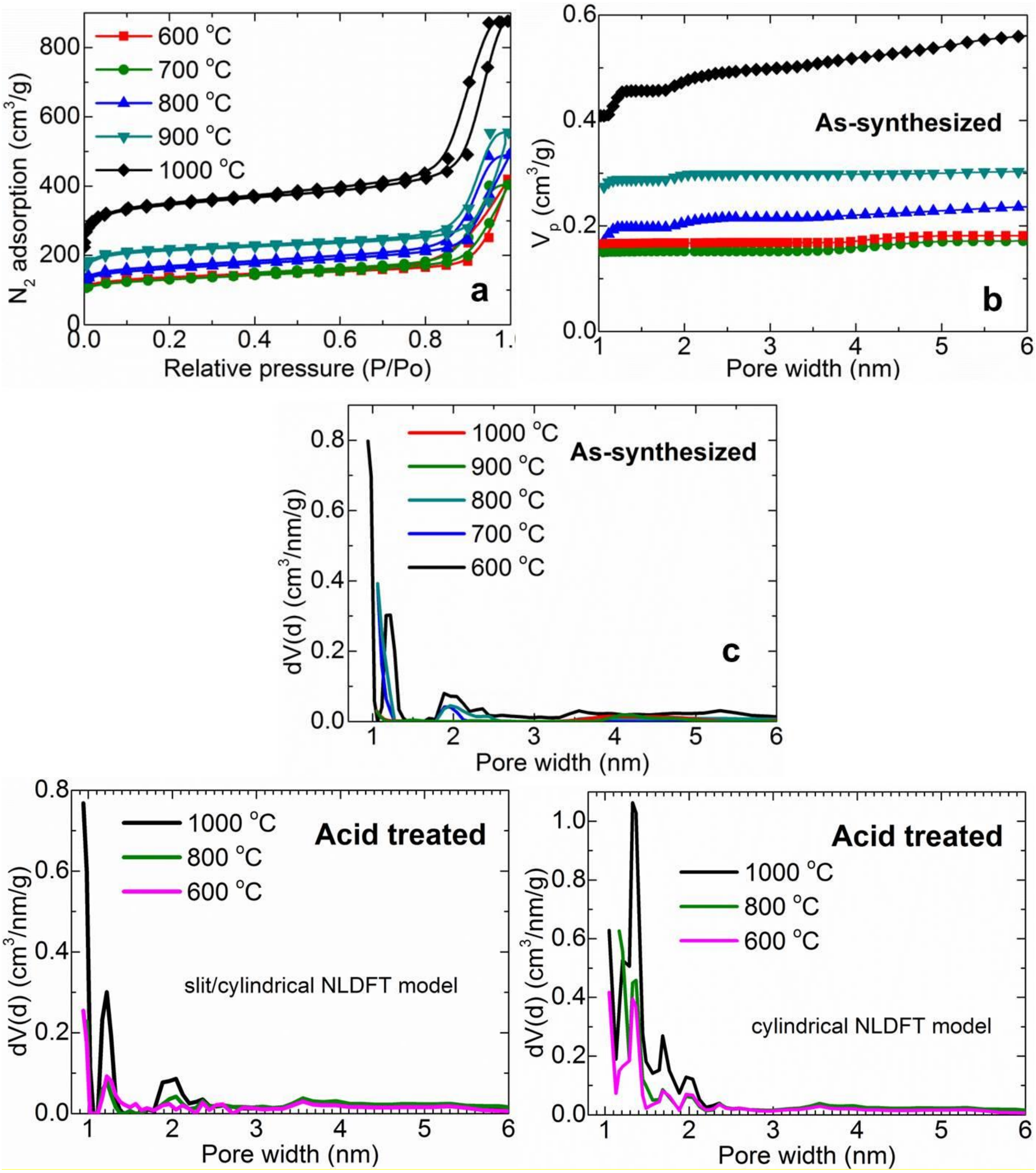

Figure S14. Top: a) 77 K N_2 adsorption-desorption isotherms, b) cumulative pore volume plots and c) pore-size distribution plots of the as-synthesized carbon samples, at different carbonization temperatures.

Bottom: Pore-size distribution plots of acid treated samples derived by applying a slit/cylindrical NLDFT (left) and cylindrical NLDFT (right).

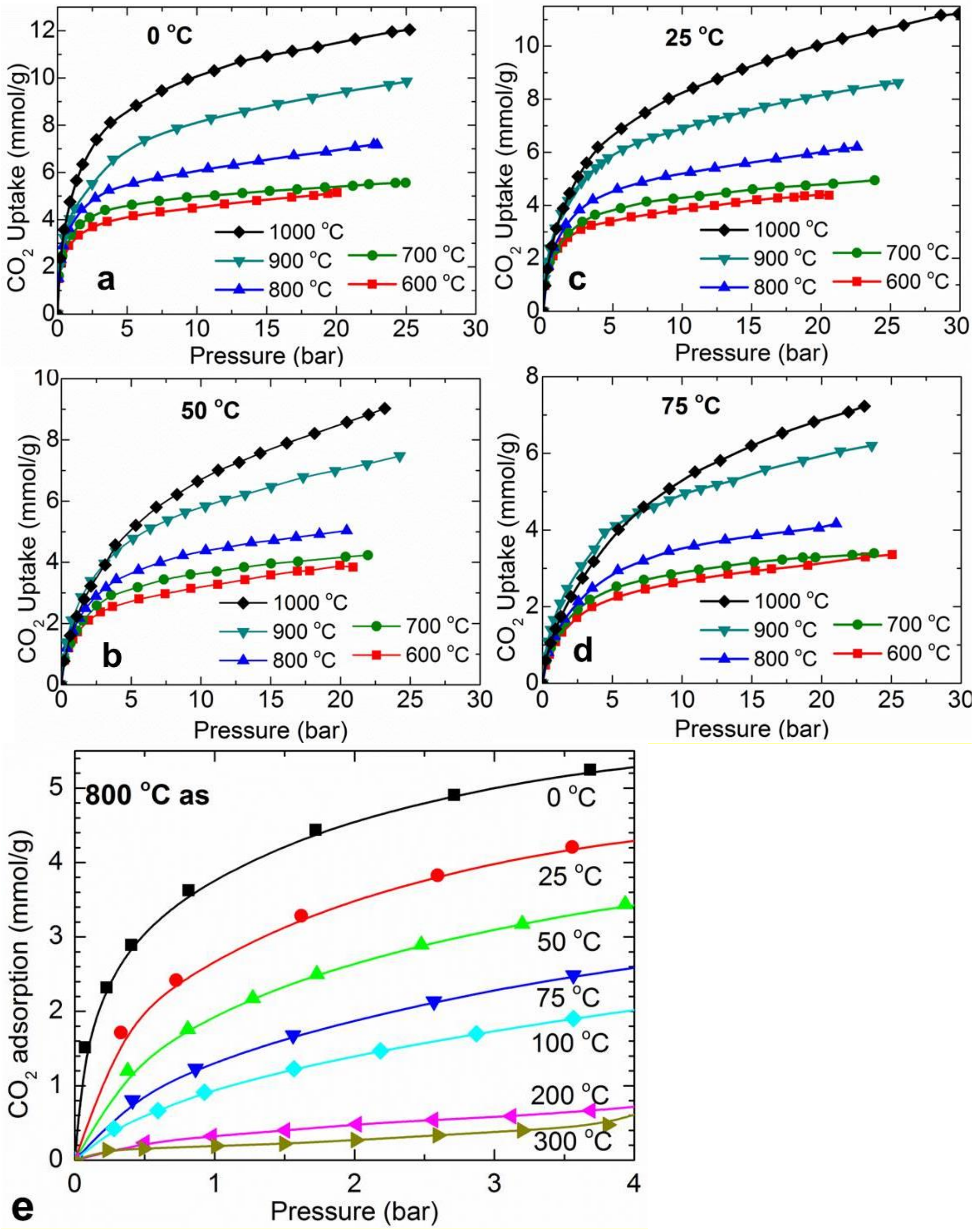

Figure S15. Top four pane: 0 °C (a), 25 °C (b), 50 °C (c) and 75 °C (d) high-pressure CO_2 uptake isotherms (up to 30 bar) of as-synthesized carbons, derived at various carbonization temperatures between 600-1000 °C. e) The CO_2 uptake isotherms between (0-300) °C in as synthesized nZDC800.

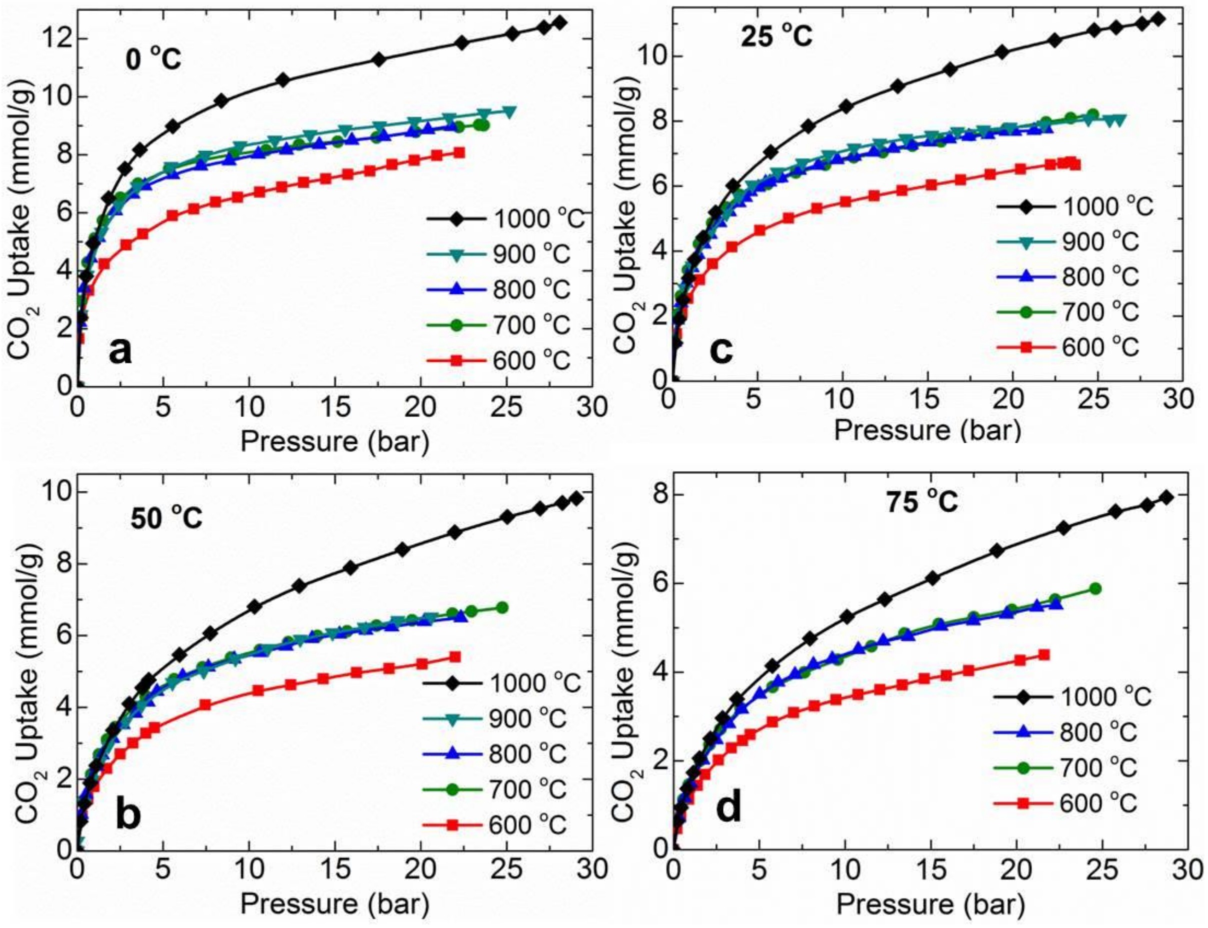

Figure S16. 0 °C (a), 25 °C (b), 50 °C (c) and 75 °C (d) high-pressure CO_2 uptake isotherms (up to 30 bar) of acid-treated carbons.

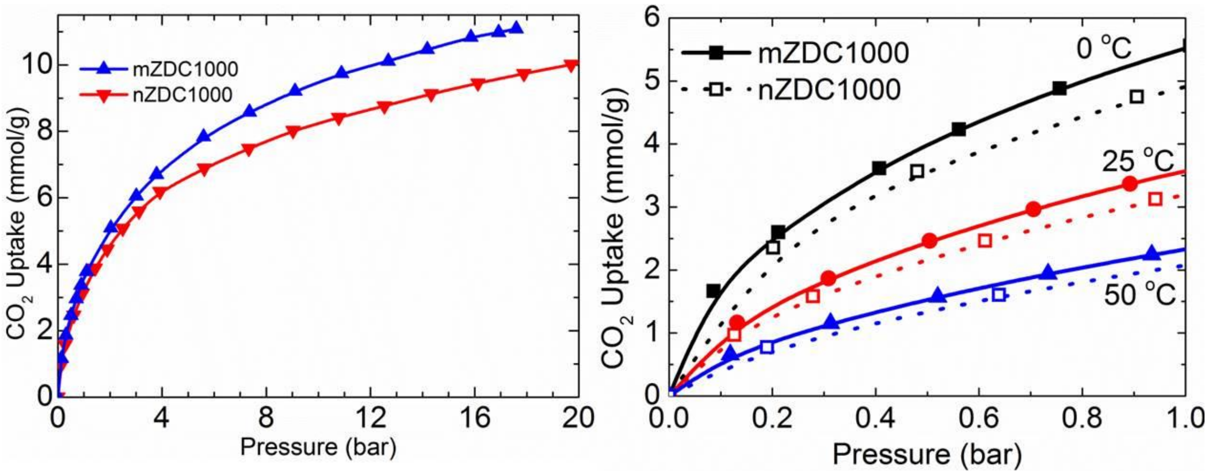

Figure S17. A comparative high- (left) and low- (right) pressure CO_2 uptake isotherms at 0°C, 25 °C and 50 °C of mZIF-8 and nZIF-8 derived carbons at same carbonization temperature of 1000 °C and residence time of 6 h.

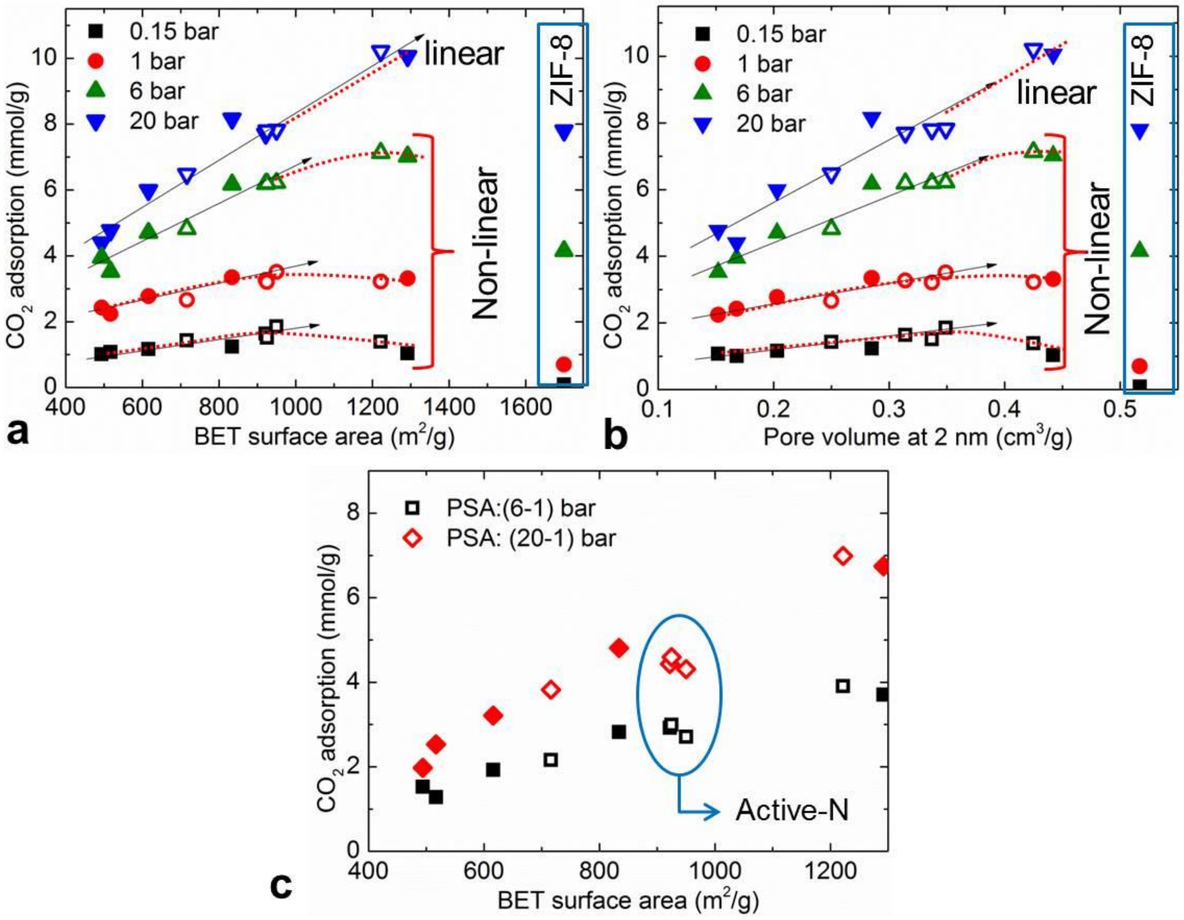

Figure S18. 25 °C CO_2 uptakes at 0.15, 1.0, 6.0, and 20 bar pressures again BET specific surface area (a) and micropore volume at 2 nm (b). c) PSA values against BET specific surface area. In all the plots the solid and open data symbols represent as-synthesized and acid-treated carbons.

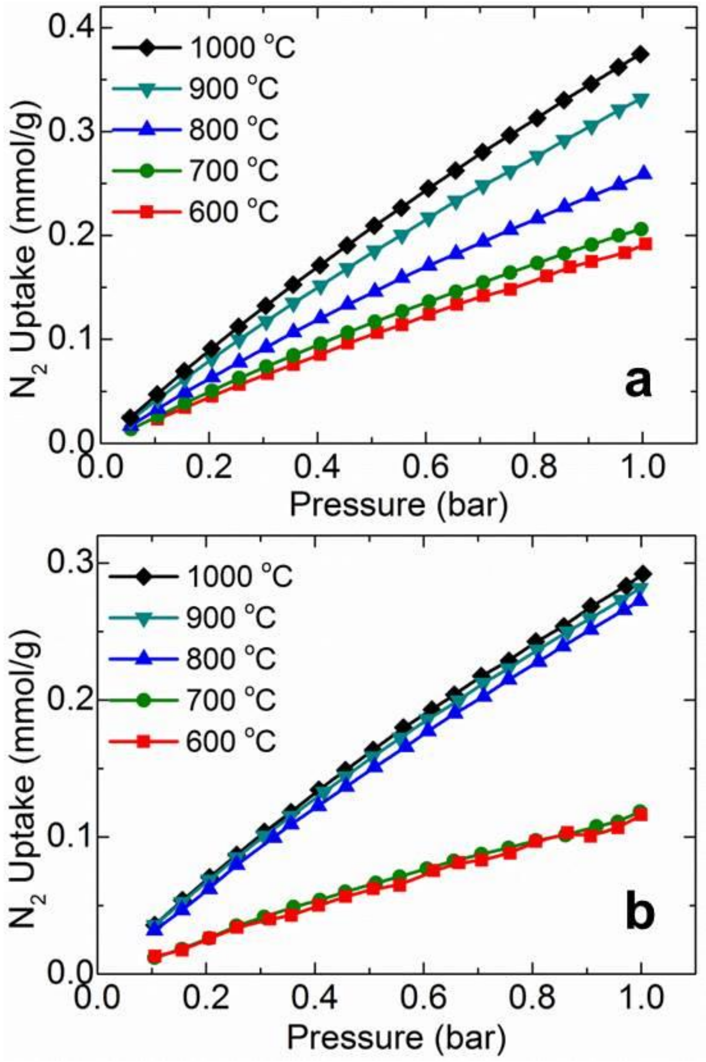

Figure S19. 25 °C N_2 uptake isotherms up to 1 bar, as-synthesized and acid-treated carbon samples are shown in (a) and (b), respectively.

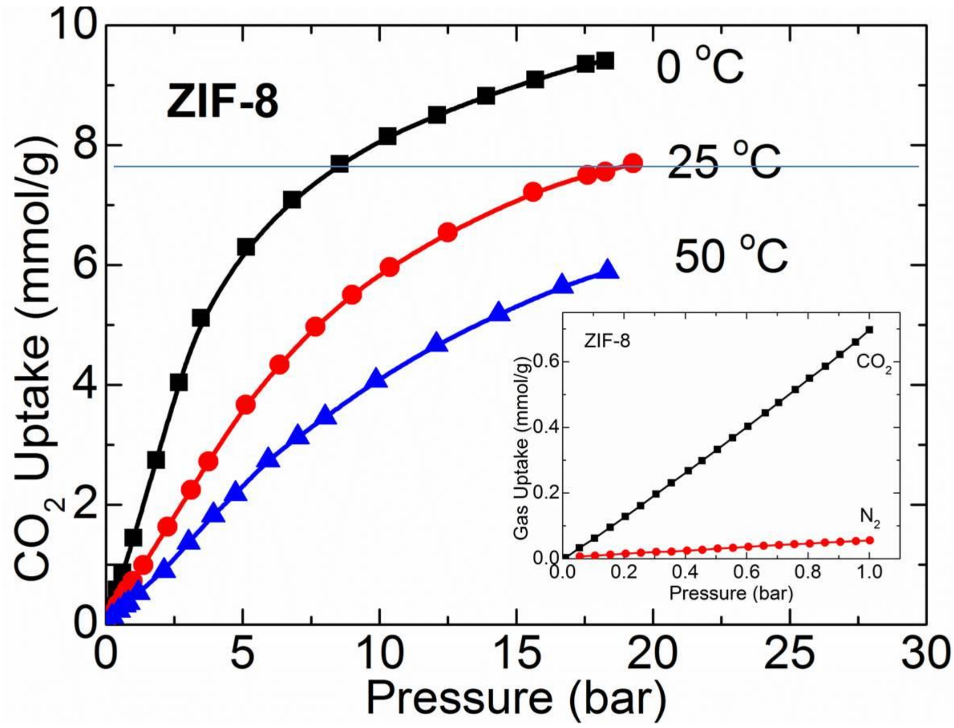

Figure S20. High & low (Inset) pressure CO_2 uptake isotherms of initial ZIF-8, measured at 0 °C, 25 °C and 50 °C. Inset also shows the N_2 uptake isotherm at 25 °C.

Table S1. Normalised room temperature CO_2 uptakes with respect to their BET surface area and micropore volume. –as and –cln stands for as-synthesized and acid clean

Sample	CO_2 uptake at 25 °C per BET SSA (μmol/m^2)				CO_2 uptake at 25 °C per V_{micr} (mmol/cm^3)			
	0.15 bar	1.0 bar	6.0 bar	20.0 bar	0.15 bar	1.0 bar	6.0 bar	20.0 bar
ZIF-8	0.055	0.41	2.44	4.59	0.18	1.35	8.03	15.09
600-as	2.08	4.33	6.81	9.23	7.07	14.74	23.16	31.38
700-as	2.04	4.916	8.00	8.91	6.01	14.45	23.51	26.19
800-as	1.89	4.50	7.63	9.72	5.74	13.67	23.15	29.51
900-as	1.49	4.02	7.40	9.78	4.35	11.75	21.65	28.63
1000-as	0.80	2.56	5.43	7.79	2.34	7.49	15.88	22.76
600-cln	2.00	3.71	6.73	9.05	5.72	10.63	19.28	25.92
700-cln	1.95	3.70	6.55	8.23	5.31	10.06	17.82	22.41
800-cln	1.77	3.54	6.71	8.35	5.21	10.41	19.71	24.52
900-cln	1.63	3.47	6.70	8.43	4.49	9.52	18.40	23.14
1000-cln	1.14	2.64	5.83	8.35	3.27	7.58	16.78	24.02

Table S2. The CO_2 adsorption performance of ZDCs and ZIF-8 at PSA (pressure swing adsorption) and VSA (vacuum swing adsorption) conditions for pure CO_2 (PSA: between 6 bar and 1 bar, VSA: between 1.5 bar and 0.05 bar) and flue gas CO_2 (20%; PSA: between 1.2 bar and 0.2 bar, VSA: between 0.3 bar and 0.001 bar). All are obtained from the 25 °C CO_2 adsorption isotherms.

Sample	Pure CO_2		Flue CO_2	
	PSA (mmol/g)	VSA (mmol/g)	PSA (mmol/g)	VSA (mmol/g)
ZIF-8	3.45	1.06	0.75	0.19
600-as	1.28	2.10	1.31	1.42
700-as	1.52	2.16	1.33	1.50
800-as	1.92	2.55	1.58	1.64
900-as	2.82	3.27	2.11	1.83
1000-as	3.71	3.49	2.27	1.63
600-clean	2.16	2.46	1.53	1.57
700- clean	2.71	3.45	2.15	2.04
800- clean	2.92	3.31	2.12	1.81
900- clean	2.99	3.41	2.25	1.68
1000- clean	3.91	3.59	2.39	1.56

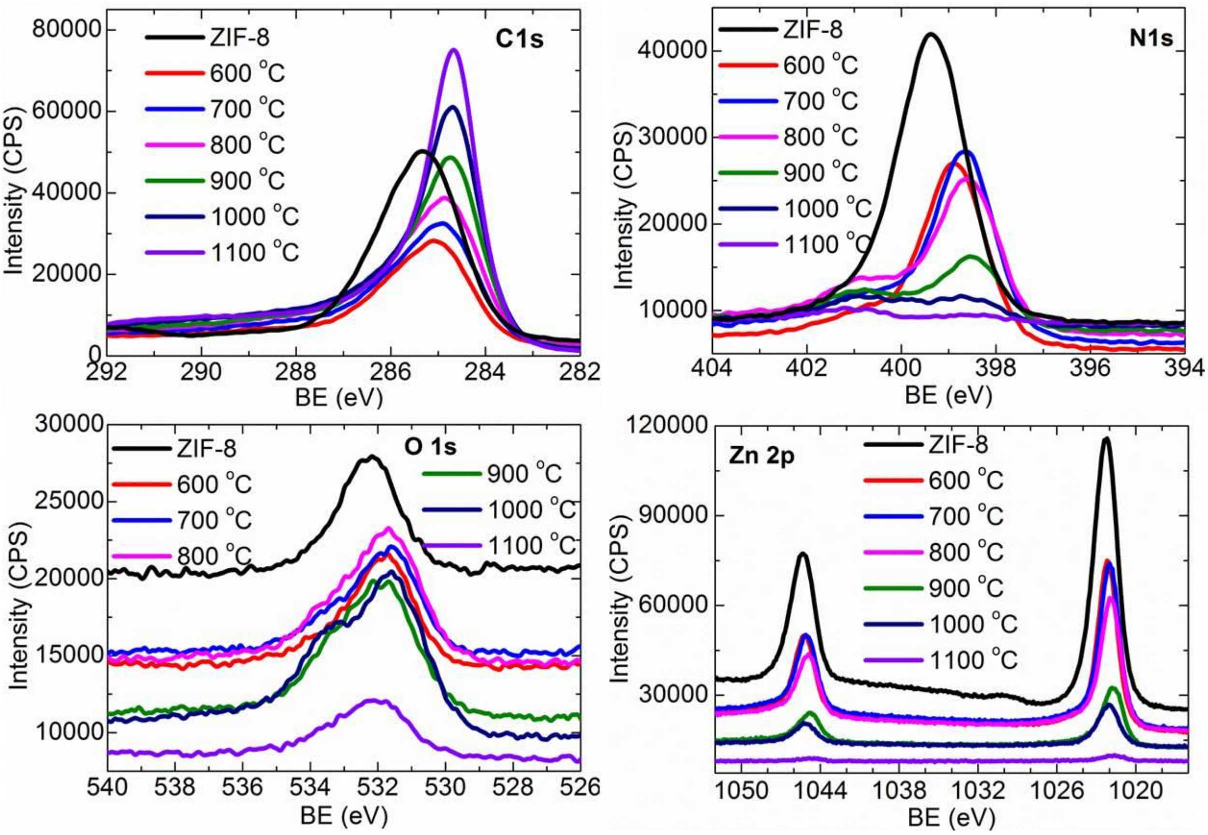

Figure 21. XPS spectra of as-synthesized nZDCs; the 4 panels starting from top-left and clockwise direction represents the core level spectra of C 1s, N 1s, Zn 2p and O 1s. The corresponding carbonization temperature is also shown on each plot with the same colour code. For understanding the ZIF-8 spectra is also included.

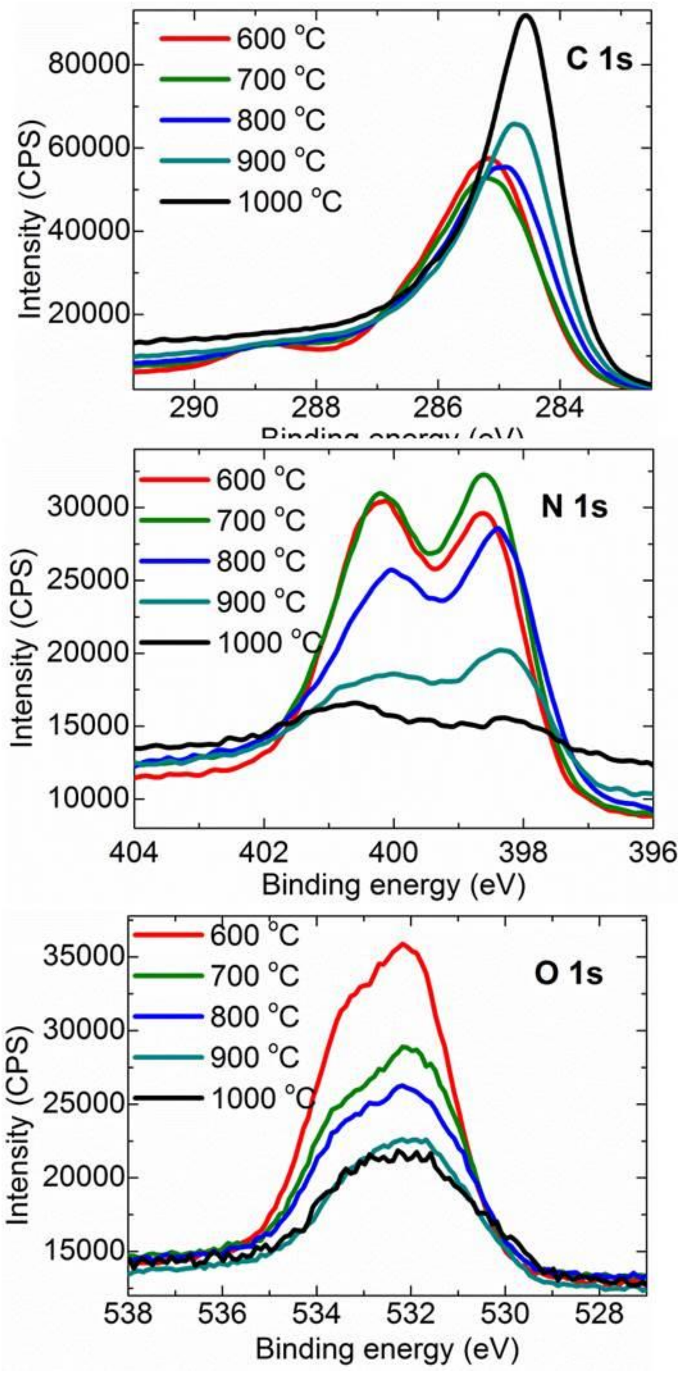

Figure 22. XPS spectra of acid-treated nZDCs; core level spectra of C 1s (a), N 1s (b) and O 1s (c). The corresponding carbonization temperature is also shown on each plot with the same colour code.

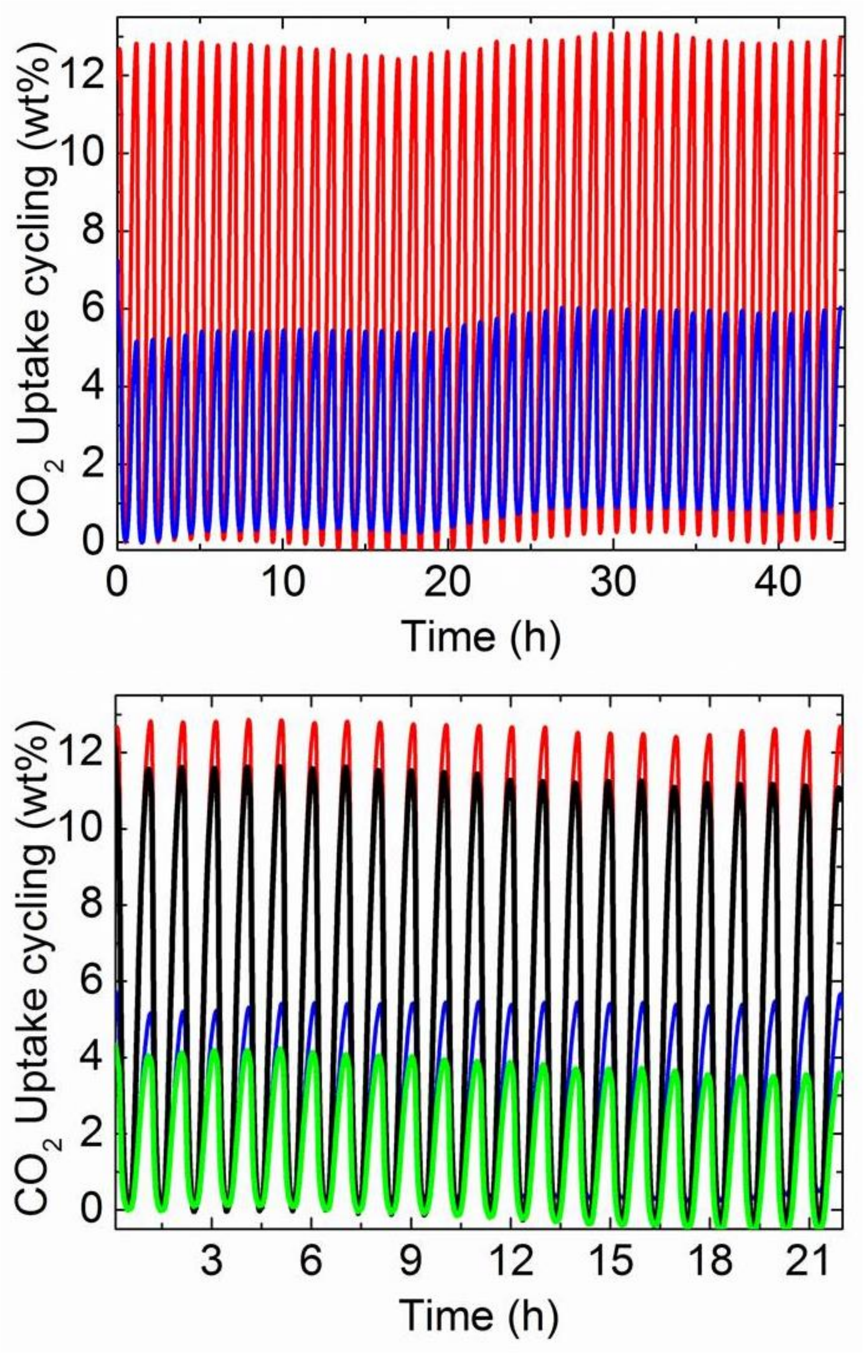

Figure 23. CO_2 cycling test on TGA under continuous flowing test gas near 1 atmosphere. The maximum uptake and release temperature set to 31 °C and 200 °C respectively. The ramp rate for heating and cooling was set to 10 & 50 °C per minute respectively. Top: up to 44 cycles test on nZDC700 sample, the red and blue line data represent the uptakes for 100% CO_2 and 15%CO_2 in 85%N_2, respectively. Bottom: A comparative 22 cycles test on nZDC1000 and nZDC700. 100%CO_2 cycling is represented by red (for nZDC700) and black (for nZDC1000) line data. 15%CO_2 in 85%N_2 cycling is represented by blue (for nZDC700) and green (for nZDC1000) line data.

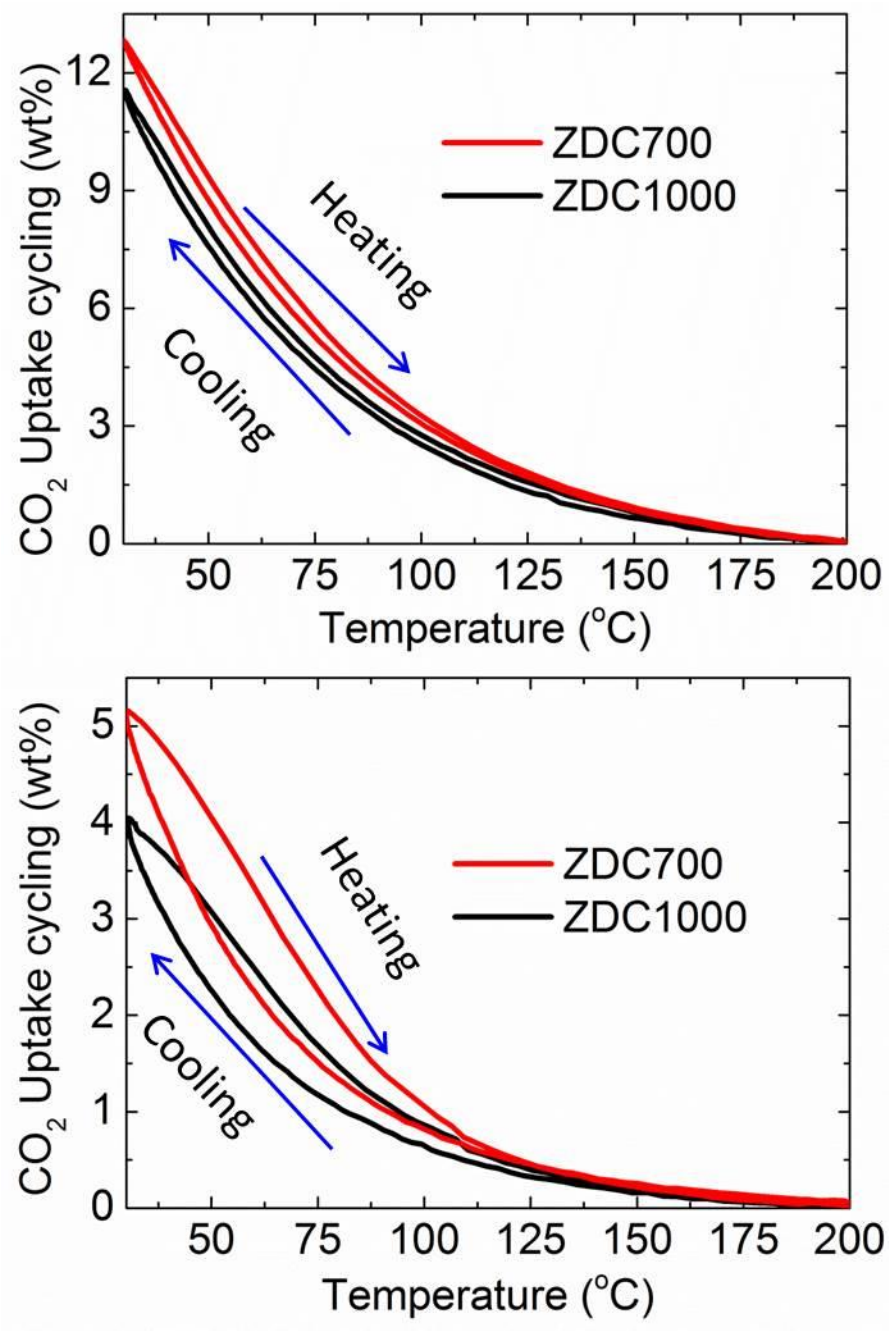

Figure 24. CO_2 uptake and release behaviour against heating (10 °C per minute) and cooling (50 °C per minute, more like natural cooling) temperature between 31 °C and 200 °C for both the samples of nZDC700 and nZDC1000. Top: 100% CO_2 flow. Bottom: for 15%CO_2 in 85%N_2 flow.

Table S3. CO_2 uptake values at 1 bar and three different temperatures of the Zn-free carbon samples obtained at different temperatures.

Sample	CO_2 uptake (mmol/g)		
	0 °C	25 °C	50 °C
nZDC-6h@600clean	3.64	2.66	1.80
nZDC-6h@700-clean	5.12	3.51	2.36
nZDC-6h@800-clean	4.79	3.27	2.14
nZDC-6h@900-clean	4.65	3.21	2.13
nZDC-6h@1000-clean	5.04	3.22	2.23
mZDC-0h@700-clean	5.34	3.28	1.46
mZDC-10h@700-clean	5.61	3.80	2.02
mZDC-24h@700-clean	5.36	3.47	1.75
mZDC-6h@1000-as	5.50	3.61	2.33

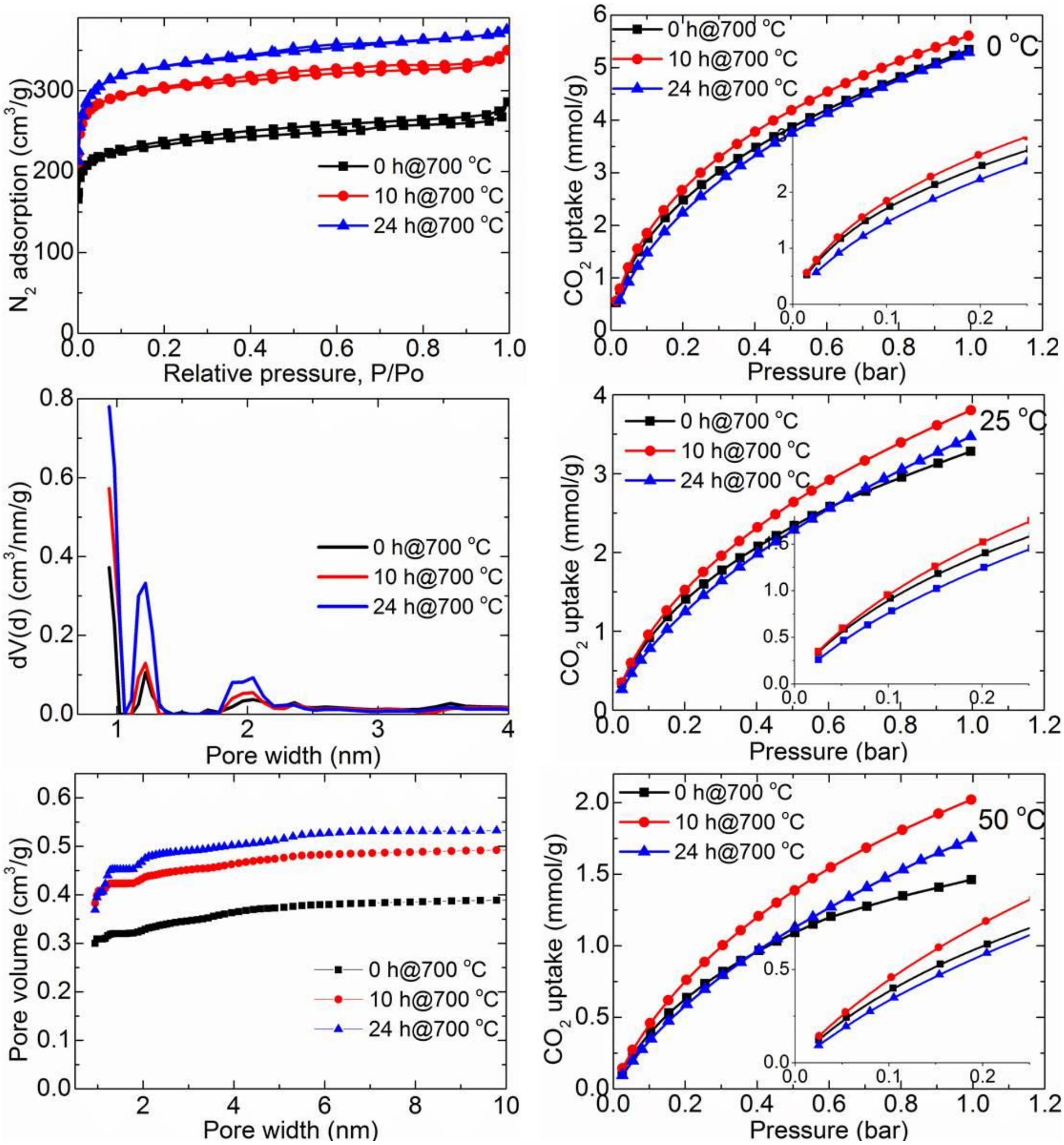

Figure S25. Porosity (left panel, top to bottom: 77 K N_2 adsorption isotherms, pore volume and poe size distribution plots) and CO_2 uptake isotherms (right panel, top to bottom: 0 °C, 25 °C and 50 °C, the insets show a very low-pressure region uptakes) of mZIF-8 derived carbons at 700 °C with different carbonization residence times of 0 h, 10 h and 24 h. Clearly, with increasing residence time, the microporosity is increased; more slit-like pores, more micropore volume and more specific surface area, e.g., 915 m^2/g at 0 h, 1190 m^2/g at 10 h and 1285 m^2/g at 24 h. In contrast, a best CO_2 uptake for the entire isotherm is seen for 10 h sample. From TGA plot in Figure S21, it can be understood that the with increasing residence time at 700 °C the sample mass loss is attributed to the further loss of C and N that leaves a more porous carbon to enhance the porosity, however, at the same time the loss of N-functional groups reduces the CO_2 uptake capacity.

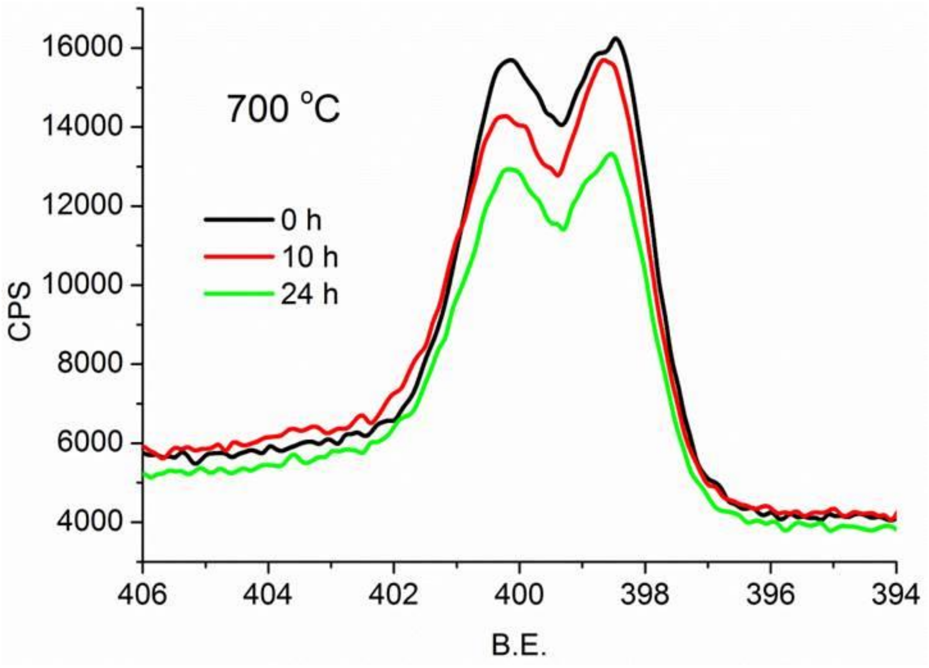

Figure S26. XPS of mZDC700 carbons with carbonization residence time of 0 h, 10 h and 24 h. A reduced N-content (ca. 23 atom% after 0 h, ca. 21 atom% after 10 h and ca. 20 atom% after 24 h) is seen with increased carbonization time.

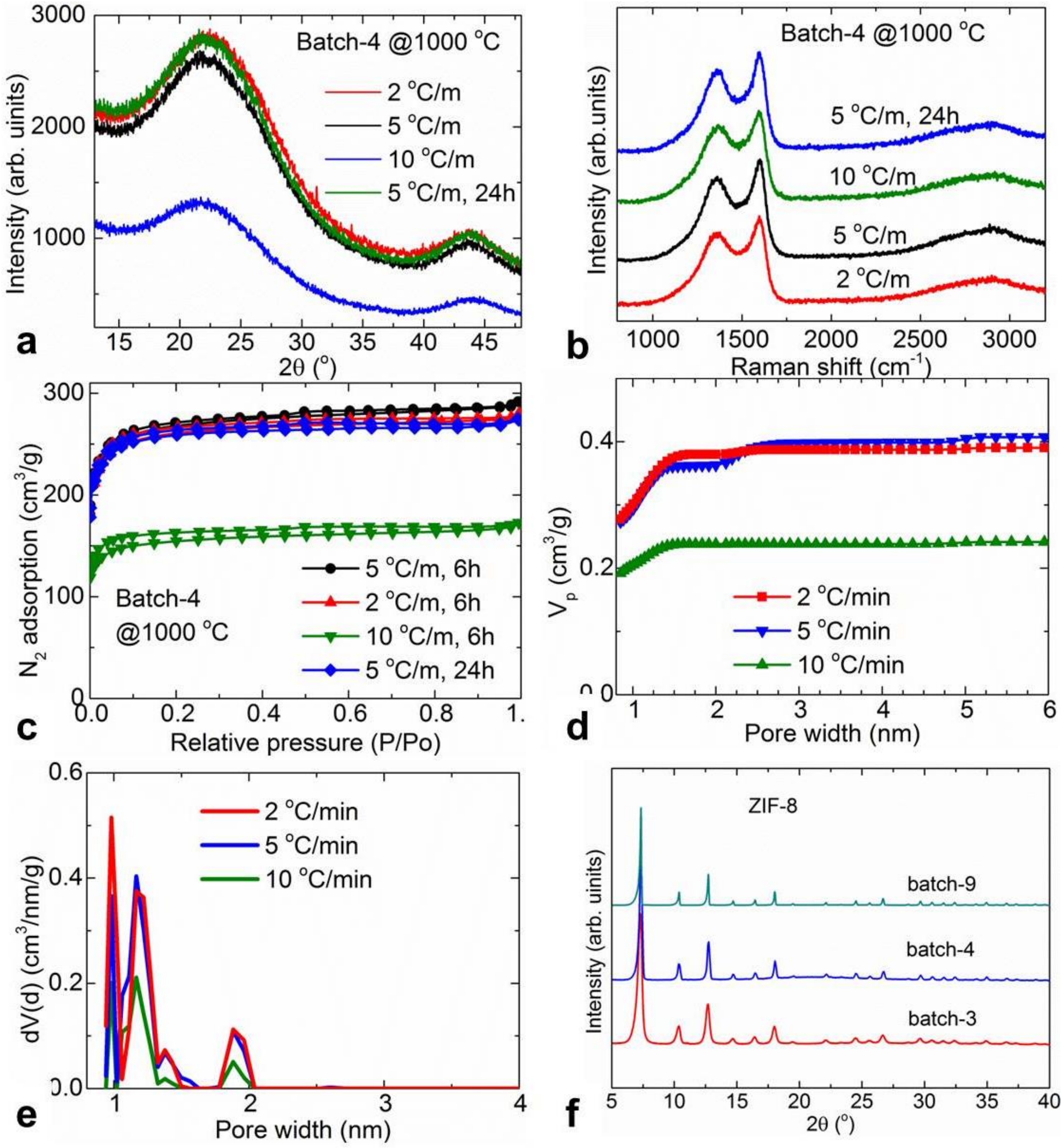

Figure S27. Structural evaluation of ZIF-8 (batch 4) carbons synthesized at 1000 °C with different carbonization conditions; heating rate (2 °C/min to 10 °C/min) and residence time (6 h and 24 h). a) PXRD patterns of ZIF-8 carbons clearly shows the more turbostratic graphitic carbon when carbonization is carried out with a heating rate of 10 °C/min. b) Raman spectra of ZIF-8 carbons. c-e) shows the porosity characteristics of ZIF-8 carbons; 77 K N_2 adsorption isotherms (c) gives BET specific surface area of 956 m^2/g, 1055 m^2/g and 609 m^2/g for the respective heating rates of 2 °C/min, 5 °C/min and 10 °C/min. The increase in carbonization residence time to 24 h at 1000 °C gives a 1026 m^2/g of surface area, much similar to the 6 h carbonization time. A clear reduction in the micropore volume and pore sized distribution is seen in (d-e). f) Shows the PXRD patterns of the ZIF-8 precursors; the broader and sharp diffraction peaks in batch 3 & 9 represents the nanosized and micron sized crystals of ZIF-8 and batch-4 shows the intermediate crystallites.

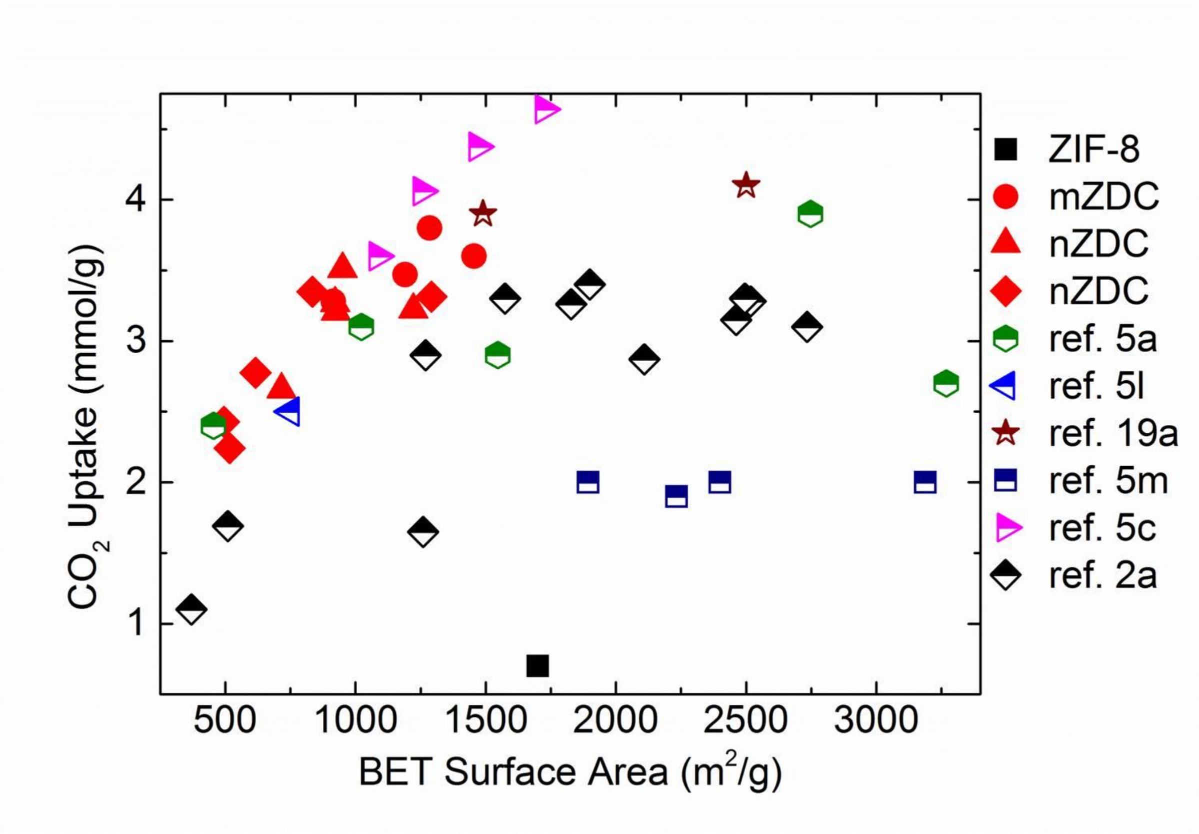

Figure S28. A comparative 25 °C CO_2 uptake values of MOF-derived carbons against BET specific surface area. Clearly as highlighted by red circles the ZDCs show better CO_2 uptake values, especially compared to other functionalized (refs. 5a, 5l, 9a-measured at 23 °C) and KOH activated (refs. 5e, 5h) ZIF-carbons and MOF-5, 74 and MIL-53(Al) derived carbons (measured at 27 °C, ref. 2a).

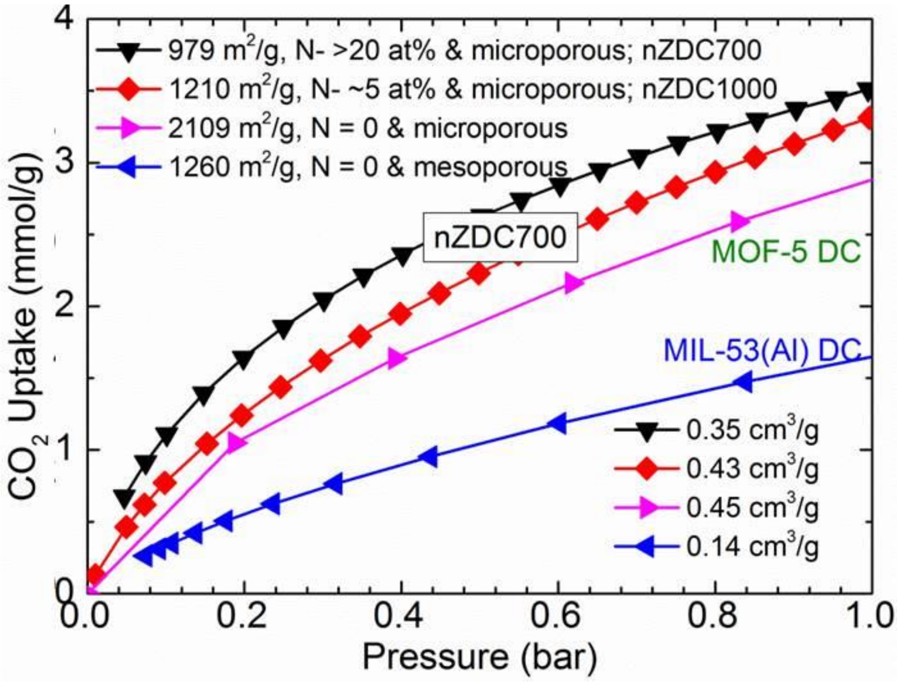

Figure S29. A comparative CO_2 uptake data of N-functionalized nZDC700, nZDC1000 with N-free pure phase carbon from MOF-5 and MIL-53(Al).[2a] The respective samples BET specific surface area in m^2/g and micropore volume in cm^3/g is given along with N-content in the carbon.

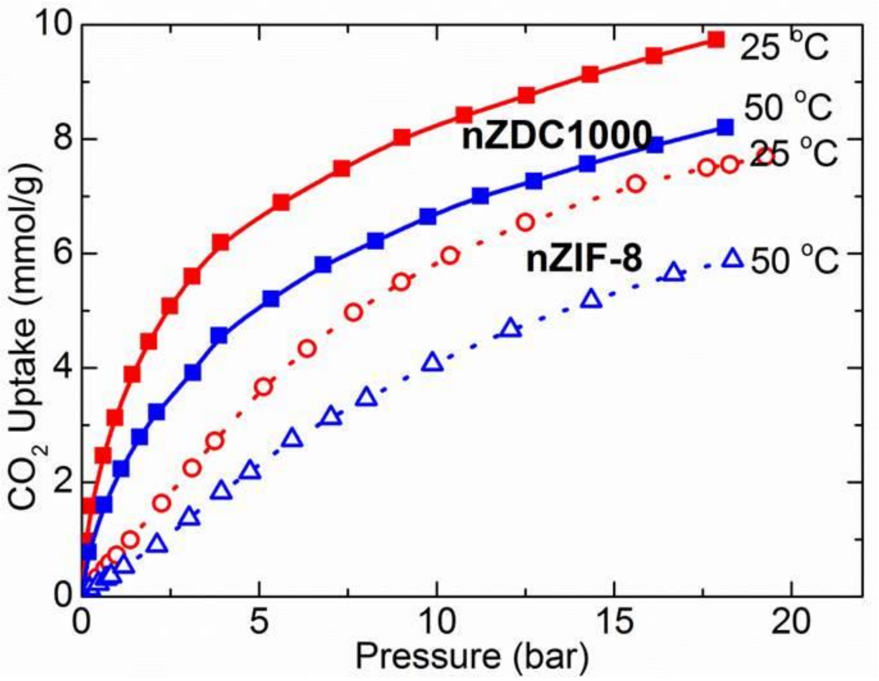

Figure S30. Comparative high-pressure CO_2 uptake isotherms measured at 25 °C and 50 °C of nZIF-8 (open data) and its carbon derivative, nZDC1000 (solid data).
